# Pharmacologic Treatment of Obesity in adults and its impact on comorbidities: 2024 Update and Position Statement of Specialists from the Brazilian Association for the Study of Obesity and Metabolic Syndrome (Abeso) and the Brazilian Society of Endocrinology and Metabolism (SBEM)

**DOI:** 10.20945/2359-4292-2024-0422

**Published:** 2024-11-25

**Authors:** Rodrigo O. Moreira, Cynthia M. Valerio, Alexandre Hohl, Cristiane Moulin, Fábio Moura, Fábio R. Trujilho, Fernando Gerchman, Livia L. Correa, Marcio C. Mancini, Maria Edna Melo, Rodrigo N. Lamounier, Simone van de Sande-Lee, Thaísa D. G. Trujilho, Paulo A. C. Miranda, Bruno Halpern

**Affiliations:** 1 Instituto Estadual de Diabetes e Endocrinologia Luis Capriglione Rio de Janeiro RJ Brasil Instituto Estadual de Diabetes e Endocrinologia Luis Capriglione, Rio de Janeiro, RJ, Brasil; 2 Centro Universitário Presidente Antonio Carlos Juiz de Fora MG Brasil Centro Universitário Presidente Antonio Carlos – Campus Juiz de Fora, Juiz de Fora, MG, Brasil; 3 Centro Universitário de Valença Valença RJ Brasil Centro Universitário de Valença, Valença, RJ, Brasil; 4 Departamento de Clínica Médica Universidade Federal de Santa Catarina Florianópolis SC Brasil Departamento de Clínica Médica, Universidade Federal de Santa Catarina, Florianópolis, SC, Brasil; 5 Centro Especializado em Diabetes, Obesidade e Hipertensão Secretaria de Saúde do Distrito Federal Brasília DF Brasil Centro Especializado em Diabetes, Obesidade e Hipertensão, Secretaria de Saúde do Distrito Federal, Brasília, DF, Brasil; 6 Universidade de Pernambuco Recife PE Brasil Universidade de Pernambuco, Recife, PE, Brasil; 7 Instituto de Medicina Integrada de Pernambuco Recife PE Brasil Instituto de Medicina Integrada de Pernambuco, Recife, PE, Brasil; 8 Centro de Diabetes e Endocrinologia da Bahia Salvador BA Brasil Serviço de Obesidade e Lipodistrofia, Centro de Diabetes e Endocrinologia da Bahia, Salvador, BA, Brasil; 9 Departamento de Clínica Médica Faculdade de Medicina Universidade Federal do Rio Grande do Sul Porto Alegre RS Brasil Departamento de Clínica Médica, Faculdade de Medicina, Universidade Federal do Rio Grande do Sul, Porto Alegre, RS, Brasil; 10 Hospital de Clínicas de Porto Alegre Porto Alegre RS Brasil Serviço de Endocrinologia e Metabolismo, Hospital de Clínicas de Porto Alegre, Porto Alegre, RS, Brasil; 11 Hospital das Clínicas Faculdade de Medicina Universidade de São Paulo São Paulo SP Brasil Grupo de Obesidade, Disciplina de Endocrinologia e Metabolismo, Hospital das Clínicas, Faculdade de Medicina, Universidade de São Paulo, São Paulo, SP, Brasil; 12 Departamento de Clínica Médica Universidade Federal de Minas Gerais Belo Horizonte MG Brasil Departamento de Clínica Médica, Universidade Federal de Minas Gerais, Belo Horizonte, MG, Brasil; 13 Hospital Mater Dei Belo Horizonte MG Brasil Serviço de Endocrinologia, Hospital Mater Dei, Belo Horizonte, MG, Brasil; 14 Santa Casa da Misericórdia de Belo Horizonte Belo Horizonte MG Brasil Serviço de Endocrinologia e Metabolismo, Santa Casa da Misericórdia de Belo Horizonte, Belo Horizonte, MG, Brasil; 15 Centro de Obesidade Hospital Nove de Julho São Paulo SP Brasil Centro de Obesidade, Hospital Nove de Julho, São Paulo, SP, Brasil

**Keywords:** Obesity, antiobesity drugs, weight loss

## Abstract

Pharmacological treatment of obesity is passing through many changes in the last decades; different agents have been approved, and newer options are leaning towards higher efficacy and a more favourable safety profile; however, medications approved for a longer time are still available and useful for many patients. This document is an 2024 Update Position Statement of Specialists from the Brazilian Association for the Study of Obesity and Metabolic Syndrome (Abeso) and the Brazilian Society of Endocrinology and Metabolism (SBEM), with the aim of reviewing all the approved medications for the management of obesity in Brazil (sibutramine, orlistat, liraglutide, semaglutide and bupropion/naltrexone fixed dose), with the addition of tirzepatide, that is approved in other countries and likely approved soon in Brazil. The review is focused on efficacy, safety profile and the impact of drugs (based on existing studies) on different comorbidities.

## INTRODUCTION

Obesity is a chronic and recurrent disease that causes or aggravates more than two hundred other diseases and is associated with increased morbidity, disability, and mortality. Following international epidemiological trends, data from the Brazilian Institute of Geography and Statistics (IBGE) show that obesity already affects a quarter of the adult population in Brazil; with its galloping rates, projections indicate that by 2035 up to 40% of the Brazilian population could be in the obesity range (1). In this scenario, it is unquestionable that every health professional must understand obesity, as even if they do not treat it directly, they will evaluate and treat people living with this disease. Welcoming the patient appropriately and discussing the consequences and therapeutic options for obesity and its related comorbidities in each consultation is essential.

The pharmacologic treatment of obesity is undergoing a transitioning period but remains extremely stigmatized. Several reasons contributing to this stigmatization have been addressed in an editorial (2); among them, the stigma of obesity itself stands out, as it is still viewed as solely dependent on “lifestyle.” Data shows that only 1% of individuals for whom medications for obesity are clinically recommended actually receive them, and many who do not have such recommendations end up using them for aesthetic purposes (3,4).

Recently, the Brazilian Association for the Study of Obesity and Metabolic Syndrome (Abeso) and the Brazilian Society of Endocrinology and Metabolism (SBEM) produced a joint document emphasizing the importance of language in reducing stigma (5). Using the term “antiobesity medications” is always recommended, while the term “weight loss drugs” should be avoided. Treating obesity is much more than “losing weight,” as it includes maintaining the lost weight and the benefits beyond weight loss. Additionally, “losing weight” can be desired by anyone, not just those with a chronic disease.

In this context, an important point that emerges is how pharmacologic treatments developed following the standards of evidence-based medicine, with approval from regulatory agencies and well-conducted clinical studies, are often confused and interchanged with treatments that have no scientific support and are potentially dangerous – from manipulated formulas to herbs sold on the internet, serums, and injections produced with no regard for sanitary or health concerns. Much of the stigma surrounding treatment has also been caused by older medications known for their poor risk-benefit ratio and various medications that have been discontinued in recent decades due to unacceptable side effects. An additional reason is the low efficacy of some drugs that had outcomes falling short of those desired by both physicians and patients.

Thankfully, advancements in pharmacologic treatments have overcome previously described barriers (6). New medications, some already available on the market and others still under investigation, have the potential to achieve clinically relevant weight losses. Outcome studies show that their therapeutic targets extend far beyond weight loss, with clear objectives of improving health indicators and quality of life. The present document aims to compile the existing evidence on antiobesity medications already approved by the National Health Surveillance Agency (Anvisa), along with their main efficacy and safety data.

Despite many advances, we are still far from ensuring proper access to medications for all individuals with obesity. The high cost of some medications is still a major barrier. The development of study protocols showing clear benefits and optimal pharmacoeconomic profiles may facilitate the future inclusion of some of these medications in the Unified Health System (SUS).

Considering all the above, it becomes quite clear that Abeso and SBEM have the duty of producing a document explaining the therapeutic options approved by Anvisa for the treatment of obesity, to guide specialists and nonspecialists toward serious and ethical treatment and distance patients from dangerous, ineffective, and expensive treatments, which remain so common in our country.

It is important to emphasize that obesity treatment goes far beyond medications, with lifestyle changes (LSCs) remaining the cornerstone. However, this document focuses on pharmacologic treatment, aiming to familiarize the reader with therapeutic options along with their effects on body weight, metabolic effects, and side effects. The treatment of obesity is complex, and many of its nuances and clinical questions will not be answered here. In the future, the two societies will collaborate on a broader and more comprehensive document to address, based on existing literature, practical questions aiming to facilitate treatment management. However, for the transformative time we are currently in, this data compilation – written by experts in the field who conducted an in-depth review of the literature for the most complete and current evidence – will serve as a guide to improve the care of people living with obesity.

## 1. SIBUTRAMINE

### 1.1 Mechanism of action

Sibutramine works by inhibiting the reuptake of norepinephrine and serotonin in the synaptic cleft and, to a lesser extent, by inhibiting the reuptake of dopamine. Its main effect is on regulating food intake, prolonging satiety rather than reducing hunger. Considering this pharmacologic characteristic, sibutramine should be classified as a satiety-inducing agent and not as an anorectic agent (7).

### 1.2 Dosage/usage instructions

Sibutramine is commercially available in 10 mg and 15 mg tablets for daily use in patients aged ≥ 18 years. The prescription must be written on a controlled B2 prescription form, accompanied by a consent form completed by the physician and the patient in triplicate, in accordance with Anvisa standards (8).

### 1.3 Tolerability/side effects

The main side effects of sibutramine are associated with its noradrenergic stimulation and sympathomimetic properties, the most common being xerostomia (29.2%), tachycardia (20.9%), constipation (18.9%), hypertension (17.5%), insomnia (17.2%), and headache (11.3%) (9).

### 1.4 Absolute contraindications

Based on the results of the Sibutramine Cardiovascular Outcomes Trial (SCOUT; described in item 1.5.6, “Effects of sibutramine on lipid metabolism”), sibutramine is contraindicated in patients with type 2 diabetes mellitus (T2DM) with at least one additional risk factor (*e.g.*, hypertension controlled by medication, dyslipidemia, active smoking, or diabetic kidney disease with evidence of microalbuminuria), coronary artery disease (CAD), stroke, arrhythmia, heart failure (HF), and inadequately controlled hypertension (levels above 145 x 90 mmHg) (10).

### 1.5 Efficacy

#### 1.5.1 Effects of sibutramine on body weight

In a systematic review of 29 clinical studies, sibutramine led to a weight loss of 2.8 kg in 12 weeks, 6 kg in 24 weeks, and 4.5 kg in 54 weeks of treatment. In studies with a duration of 44-54 weeks, the difference in the proportions of participants achieving 5% weight loss was 34% for sibutramine *versus* 19% for placebo, and for those achieving 10% weight loss, it was 31% for sibutramine *versus* 12% for placebo (11).

Sibutramine led to improvement in anthropometric measurements. A Cochrane database systematic review of studies on weight loss medications with 12-18 months of follow-up assessed the effect of sibutramine on reducing weight, waist circumference, and body mass index (BMI). Patients using sibutramine lost 4.3% more weight than those using placebo in 10 of the evaluated studies. Five studies showed a BMI reduction of 1.5 kg/m^2^, and eight studies showed a waist circumference reduction of 4.0 cm (12).

#### 1.5.2 Effects of sibutramine on weight loss maintenance

Sibutramine has also proven effective in preventing weight regain when added after dietary interventions. The double-blind, randomized controlled trial (RCT) Sibutramine Trial on Obesity Reduction and Maintenance (STORM) showed the benefits of sibutramine on weight loss and maintenance over 2 years of treatment. In the study, 605 patients with obesity received sibutramine (10 mg/day) associated with a low-calorie diet for 6 months. Patients who achieved > 5% weight loss after 6 months were allocated to continue sibutramine or switch to placebo for 18 months. The sibutramine group had greater weight loss than the placebo group at 2 years (-4.0 kg [-2.4 to -5.6 kg]), reinforcing the importance of maintaining the medication for longer after the initial weight loss (13).

Another systematic review including three studies evaluated weight loss maintenance with sibutramine. The analysis showed that after an initial dietary weight loss intervention lasting 1-6 months, individuals who achieved a body weight loss of at least 5% were randomized to treatment with placebo or sibutramine. The results demonstrated that 10%-30% more individuals treated with sibutramine compared with placebo were successful in maintaining the initial loss (defined as the maintenance of 80%-100% of the weight lost after 12-18 months of treatment) (14).

#### 1.5.3 Effects of sibutramine on body composition

The effects of sibutramine on body composition have been evaluated in very specific studies. The first RCT evaluated the effects of sibutramine 10 mg for 12 weeks in 24 adolescents. No difference in body composition was found in the sibutramine group compared with the placebo group (15). The second RCT, with the same duration as the first (12 weeks), evaluated sibutramine at doses of 10 mg and 15 mg in 181 individuals with obesity. The sibutramine group showed a trend toward a greater reduction in body fat percentage than the placebo group (p = 0.05). No difference in lean mass was observed between the groups (16).

In the STORM study, a subgroup analysis showed a preferential reduction of visceral adipose tissue compared with subcutaneous tissue in body composition assessed by computed tomography (17).

#### 1.5.4 Effects of sibutramine in patients with prediabetes/glucose intolerance

We found no studies evaluating sibutramine in this population.

#### 1.5.5 Effects of sibutramine in patients with type 2 diabetes mellitus

A meta-analysis evaluating long-duration, high-quality studies on glycemic control in patients with T2DM showed that treatment with sibutramine is associated with a slight improvement in glycemic control (18). Specifically, an RCT with a 1-year duration evaluating 195 individuals with T2DM and daily sibutramine doses of 15 mg and 20 mg showed that patients using this medication experienced improved glycemic control in parallel with weight loss. Individuals with a 10% weight loss showed an average 1.2% reduction in glycated hemoglobin (HbA1c) level (19).

#### 1.5.6 Effects of sibutramine on lipid metabolism

A meta-analysis of 29 studies showed that treatment with sibutramine is associated with a slight improvement in low-density lipoprotein cholesterol (LDL-c) and triglyceride levels (18). Sibutramine was associated with a reduction in triglyceride levels, in addition to a slight increase in high-density lipoprotein cholesterol (HDL-c) levels compared with placebo, with better results observed in patients with greater weight loss (18). Another meta-analysis showed improvement in triglyceride levels compared with placebo (-7.7 mmol/L) and an increase in HDL-c (1.5 mg/L), while LDL-c levels did not differ between groups (14).

#### 1.5.7 Effects of sibutramine on blood pressure and heart rate

In a meta-analysis conducted by Rucker and cols., seven of the included studies showed that the use of sibutramine was associated with an increase in systolic (mean 1.7 mmHg, from 0.1 to 3.3 mmHg) and diastolic (mean 2.4 mmHg, from 1.5 to 3.3 mmHg) blood pressure (BP) and heart rate (mean 4.5 bpm, from 3.5 to 5.6 bpm) (14). Despite the observed increase in BP levels, BP control in individuals with previously controlled hypertension was not compromised when their antihypertensive medication was adjusted (20).

Therefore, sibutramine must be used with regular heart rate and BP monitoring. Patients who present a significant increase in these parameters should have their treatment discontinued. Of note, the treatment can be carried out as long as the absolute contraindications are respected (item 1.4, “Absolute contraindications”).

#### 1.5.8 Effects of sibutramine on obstructive sleep apnea syndrome

An uncontrolled study of 87 patients with obesity using sibutramine 10 mg/day for 6 months showed that this treatment associated with LSCs resulted in a weight loss of 8.3 ± 4.7 kg, which was accompanied by reductions in neck circumference, obstructive sleep apnea syndrome (OSAS) severity (decrease in apnea-hypopnea index [AHI] of 16.3+/-19.4 events/h), and Epworth sleepiness scale score (decrease of 4.5+/-4.6) (21).

A study compared the efficacy of weight loss induced by sibutramine *versus* continuous positive airway pressure (CPAP) treatment over a 1-year period in 40 patients with obesity, specifically evaluating their effects on sleep respiratory parameters. The sibutramine group had a body weight decrease of 5.4 ± 1.4 kg compared with the CPAP group, which had no weight loss. Treatment with CPAP improved all respiratory and sleep parameters, while sibutramine-induced weight loss improved only the nocturnal profile of arterial oxygen saturation (22).

#### 1.5.9 Effects of sibutramine in patients with polycystic ovary syndrome

An RCT including 40 women with obesity and polycystic ovary syndrome (PCOS) evaluated the efficacy of sibutramine therapy alone or combined with ethinylestradiol-cyproterone (EE-CPA) on clinical and metabolic parameters in women with obesity and PCOS. At the end of the study, there were significant decreases in BMI value, Ferriman-Gallwey hirsutism score, and serum total testosterone, free testosterone, and dehydroepiandrosterone sulfate (DHEAS) levels and a significant increase in sex hormone-binding globulin (SHBG) level in both groups. The sibutramine group had a greater reduction in body weight, waist/hip ratio, diastolic BP (DBP), and triglyceride levels, along with improvement in insulin sensitivity, which are important pathophysiological factors in PCOS (23).

A small, open-label, randomized study included 59 women with overweight or obesity and PCOS treated with sibutramine 10 mg for 6 months. The women were divided into a group on a low-calorie diet plus sibutramine and another group on a low-calorie diet alone. Body weight decreased in both groups, but the decrease was greater with sibutramine. In both groups, all women with an abnormal oral glucose tolerance test (OGTT) at baseline had normal glucose tolerance at 6 months. The free androgen index, glucose area under the curve, and fasting triglyceride level decreased at 6 months only in the group using sibutramine (24).

#### 1.5.10 Effects of sibutramine in patients with male hypogonadism

In a case report of a patient with a mutation in the melanocortin 4 receptor (MC4R) who had obesity and hypogonadotropic hypogonadism and was experiencing increasing body weight, sibutramine led to the maintenance of body weight and improved body composition and metabolic abnormalities related to obesity (25).

#### 1.5.11 Effects of sibutramine on metabolic dysfunction-associated steatotic liver disease

Only one small study has evaluated the effects of sibutramine on metabolic dysfunction-associated steatotic liver disease (MASLD). Thirteen individuals with obesity and nonalcoholic steatohepatitis (NASH) were evaluated over a 6-month period. There was a 10.2% decrease in body weight, along with a reduction of 47% in insulin resistance and declines of 41% in AST, 59% in ALT, and 27% in gamma-glutamyl transferase levels. Ultrasonographic regression of steatosis was observed in 11 of 13 patients using sibutramine. The study concluded that sibutramine-induced weight loss reduced insulin resistance and improved biochemical markers and ultrasonographic findings in patients with NASH (26).

#### 1.5.12 Effects of sibutramine on quality of life

A pooled analysis of four RCTs evaluated 555 patients with obesity regarding the impact of sibutramine treatment on quality of life assessed by the Impact of Weight on Quality of Life (IWQOL) and Short Form Health Survey (SF-36) scales. The SF-36 scale is a questionnaire with 36 questions that cover several dimensions of physical and mental health, including physical function, pain, vitality, and mental health, among other aspects. The IWQOL questionnaire assesses health-related quality of life specifically in patients with obesity. It comprises 31 questions evaluating patients’ perceptions of their weight, self-esteem, social life, physical activity, physical comfort, and other quality-of-life aspects. The study found that weight loss in the sibutramine group led to a significant improvement in quality of life, with the improvement being proportional to the weight loss (27).

#### 1.5.13 Effects of sibutramine on osteoarticular diseases

We found no studies evaluating sibutramine in this population.

#### 1.5.14 Effects of sibutramine in patients with chronic kidney disease

We found no studies evaluating sibutramine in this population.

#### 1.5.15 Effects of sibutramine on cardiovascular diseases

The SCOUT study assessed cardiovascular outcomes in individuals with cardiovascular disease (CVD; coronary artery disease, stroke, or peripheral arterial occlusive disease), T2DM with one or more cardiovascular risk factors, or both. In patients treated with sibutramine, the risk rate of cardiovascular events increased by 16%, with the risk of nonfatal acute myocardial infarction (AMI) increasing by 28% and the risk of nonfatal stroke increasing by 36%. Thus, sibutramine was associated with an increased risk of nonfatal cardiovascular events in this group of patients. The risks of death from cardiovascular causes and cardiorespiratory arrest were not different between the two groups (28).

## 2. ORLISTAT

### 2.1 Mechanism of action

Orlistat acts in the gastrointestinal tract by decreasing the absorption of dietary fats. Its mechanism of action involves irreversibly inhibiting gastric and pancreatic lipases, which reduces the hydrolysis of triglycerides into fatty acids and monoglycerides and decreases the absorption of ingested fat by 30% (29,30).

### 2.2 Dosage/usage instructions

The recommended daily dose of orlistat is 120 mg to be taken during or up to 1 hour after each of the three main meals (29).

### 2.3 Tolerability/side effects

The main side effects associated with orlistat therapy are related to the gastrointestinal system. A meta-analysis including 16 studies found that over 80% of patients treated with orlistat had at least one gastrointestinal side effect (12). The most frequent gastrointestinal side effects were steatorrhea, urgency to defecate, and flatulence with fat elimination, each with frequency rates of 15%-30% in most studies (14). Notably, the effects of diarrhea and abdominal pain are commonly observed in individuals with low adherence to diet (31).

Due to its effect on reducing the absorption of intestinal fat, chronic use of orlistat results in decreased absorption of fat-soluble vitamins (A, D, E, and K) (30). Additionally, dietary fat can bind to calcium in the intestinal lumen, leading to increased intestinal absorption of oxalate and preventing oxalate from naturally binding with intraluminal oxalate. The increase in circulating oxalate can lead to hyperoxaluria, a condition associated with the formation of kidney stones (32,33).

### 2.4 Absolute contraindications

Orlistat is contraindicated in pregnant and breastfeeding women. Patients with chronic malabsorption syndrome or cholestasis should also not use this medication (34).

### 2.5 Efficacy

#### 2.5.1 Effects of orlistat on body weight

A meta-analysis including RCTs with a duration of at least 1 year and the use of orlistat 120 mg three times daily found greater weight reductions in the orlistat group compared with the placebo group. Patients treated with orlistat lost 2.9 kg (2.5-3.2 kg) more weight than those treated with placebo. A greater number of participants in the orlistat group achieved clinically significant weight loss, with 21% and 12% achieving 5% and 10% of body weight loss, respectively. The same study showed a greater reduction in waist circumference with orlistat therapy (2.06 cm) compared with placebo (12).

#### 2.5.2 Effects of orlistat on body weight maintenance

The Xenical in the Prevention of Diabetes in Obese Subjects (XENDOS) study was designed to evaluate the prevention of diabetes in individuals with prediabetes using orlistat. This 4-year RCT included 3,305 patients with obesity and without a T2DM diagnosis who had normal glucose tolerance or were intolerant to oral glucose. Intensive LSCs were recommended, associated with treatment with orlistat or placebo. Weight loss was significantly greater with orlistat than with placebo at 1 year (10.6 *versus* 6.2 kg, respectively) and remained significantly greater at the end of the fourth year of the study (5.8 *versus* 3.0 kg, respectively) (35).

#### 2.5.3 Effects of orlistat on body composition

Two small studies evaluated the effects of orlistat on body composition. The first, an RCT, compared the effects of 1 year of treatment with orlistat or placebo on body composition assessed by dual-energy X-ray absorptiometry (DXA). Interestingly, weight loss was significant in both the orlistat and placebo groups, but there was no significant difference between the two groups (11.2 ± 7.5 kg *versus* 8.1 ± 7.5 kg). There was also no significant difference between groups in relation to body composition parameters (fat-free mass [FFM], fat mass [FM], or percentage fat mass [FM%]), although both groups showed reductions in these three parameters (36). The second study included 72 patients who completed a 2-year RCT comparing orlistat *versus* placebo. Body composition (FM and FFM) was assessed using bioimpedance, and the FM/FFM ratio was calculated. After a 12-month period, the groups had a significant reduction in FFM, but the difference between the two groups was not significant. In contrast, patients in the orlistat group had a greater reduction in FM (38.0 ± 7.6 kg to 29.1 ± 11.2 kg) than those in the placebo group (37.5 ± 8.5 kg to 32.3 ± 11.2 kg) (37).

#### 2.5.4 Effects of orlistat in patients with prediabetes/glucose intolerance

The effects of orlistat in individuals with prediabetes were evaluated in the XENDOS study (described previously). After 4 years of treatment, the cumulative risk of developing diabetes was 9.0% in the placebo group and 6.2% in the orlistat treatment group, corresponding to a risk reduction of 37.3%. Among 21% of the individuals with impaired glucose tolerance at baseline, the incidence of T2DM decreased by 45.0% over 4 years of orlistat therapy (35).

#### 2.5.5 Effects of orlistat on glycemic control in patients with type 2 diabetes mellitus

A meta-analysis included 2,550 patients with obesity and T2DM who used orlistat 120 mg three times daily or placebo. Weight loss was 2.4 kg greater in the orlistat group than in the placebo group. Patients treated with orlistat had significantly greater reductions in mean fasting plasma glucose and HbA1c levels than those treated with placebo (1.39 mmol/L *versus* 0.47 mmol/L and 0.74% *versus* 0.31%, respectively) (38).

A systematic review analyzed 12 RCTs of orlistat associated with LSCs in individuals with T2DM. Orlistat, compared with lifestyle interventions alone, led to a greater mean weight loss (2.10 kg) (39).

A subgroup analysis of patients with T2DM from five studies included in a meta-analysis of orlistat for weight loss showed a 2.3% weight reduction, along with decreases in fasting blood glucose by 1.0 mmol/L (95% CI = 0.6-1.5 mmol/L) and HbA1c levels by 0.4% (95% CI = 0.2%-0.6%) (12).

#### 2.5.6 Effects of orlistat on lipid metabolism

A systematic review and meta-analysis evaluated the effects of orlistat on different lipid profile parameters. It included 13 studies assessing the effects of orlistat on total cholesterol (n = 5,206), 13 studies with effects on LDL-c (n = 5,206), 11 studies with effects on HDL-c (n = 4,152), and 11 studies with effects on triglycerides (n = 4,456). The results showed that orlistat promoted average reductions of 12.4 mg/dL in total cholesterol (10.8-14.3 mg/dL), 10.05 mg/dL in LDL-c (8.5-11.6 mg/dL), and 1.1 mg/dL in HDL-c (0.77-1.5 mg/dL). No significant effects were observed on triglyceride levels (12).

A 24-week RCT evaluated the effects of orlistat 120 mg three times daily *versus* placebo on weight loss and serum lipids in patients with obesity and dyslipidemia. The mean percentage of weight loss was 6.8% in the orlistat group compared with 3.8% in the placebo group (p < 0.001). The orlistat group, compared with the placebo group, experienced a significant reduction in total cholesterol (11.9% *versus* 4.0%, respectively) and LDL-c (17.6% *versus* 7.6%, respectively; p < 0.001). For different weight reductions, the change in LDL-c level was more pronounced in the orlistat group, indicating a possible direct effect of orlistat on cholesterol reduction independent of weight reduction (40).

#### 2.5.7 Effects of orlistat on blood pressure and heart rate

A meta-analysis of 16 studies found a 1.5 mmHg (0.9-2.2 mmHg) reduction in placebo-subtracted systolic BP (SBP) in 13 studies and a 1.4 mmHg (0.7-2.0 mmHg) reduction in DBP in 12 studies (12).

#### 2.5.8 Effects of orlistat on obstructive sleep apnea syndrome

One RCT compared orlistat *versus* placebo over a 2-year period in patients with obesity. Orlistat improved the quality of life among patients with OSAS, but its effect on AHI was not measured (41). Another randomized study compared orlistat 120 mg three times daily *versus* placebo for 2 years in 743 patients with obesity. The use of orlistat to promote weight loss resulted in improved vitality among patients with OSAS, as measured by the SF-36, but the trial did not measure AHI or other sleep parameters (42).

#### 2.5.9 Effects of orlistat in patients with polycystic ovary syndrome

A meta-analysis of eight studies evaluated the use of oral contraceptives (OCP) plus orlistat compared with OCP alone in patients with PCOS with overweight or obesity. The combined OCP plus orlistat treatment was more effective than OCP alone in reducing weight and hormonal, lipid, and insulin metabolism parameters, as well as improving ovulation and pregnancy rates compared with OCP alone (43). A systemic review of six RCTs assessed the efficacy of orlistat *versus* metformin in women with obesity and PCOS and found significant reductions in weight loss along with total cholesterol and triglyceride levels in the orlistat group compared with the metformin group (44).

#### 2.5.10 Effects of orlistat in patients with male hypogonadism

There are no studies on the effects of orlistat in patients with male hypogonadism.

#### 2.5.11 Effects of orlistat on metabolic dysfunction-associated steatotic liver disease

A meta-analysis of seven studies, of which only three were RCTs, evaluated the effects of orlistat in patients with MASLD and overweight or obesity. In all, 330 patients with hepatic steatosis or steatohepatitis were evaluated. Despite the improvement in laboratory parameters (transaminases), no improvement in steatosis, steatohepatitis, or fibrosis was observed (45). A systematic review of studies assessing weight loss medications evaluated the effect of orlistat 120 mg twice daily associated with LSCs in six studies lasting at least 24 weeks and including patients with MASLD and overweight or obesity. All studies found reduced hepatic fat content and/or reduced liver enzymes (ALT and AST) concomitant with a 5%-10% reduction in body weight loss. Additionally, three studies reported improvement in histopathological findings. The results suggest that the reduction in hepatic fat content was primarily due to weight loss, with no evidence of independent effects of orlistat on MASLD (46).

#### 2.5.12 Effects of orlistat on quality of life

One RCT compared orlistat *versus* placebo over a 2-year period in patients with obesity. Patients treated with orlistat reported significantly greater satisfaction with their antiobesity medication than those receiving placebo at 1 and 2 years (p < 0.001 in the orlistat 120 mg group; p < 0.05 in the orlistat 60 mg group). Patients who used orlistat 120 mg experienced improved quality of life (47).

#### 2.5.13 Effects of orlistat on osteoarticular diseases

A 6-month RCT including 50 women aged 45-60 years with obesity and Kellgren-Lawrence stage II-III knee osteoarthritis found that weight reduction was significantly greater in patients treated with orlistat (9.05%; average 9.5 kg) than in those who only followed a hypocaloric diet (2.54%; average 2.66 kg). Body weight reduction in patients with orlistat reduced joint pain by 52% and joint stiffness by 51%, and improved joint functional insufficiency by 51% and quality of life by 52% (48).

A retrospective, non-placebo-controlled study analyzed the medical records of 10 women with overweight and knee osteoarthritis treated for 6 months with orlistat 120 mg three times daily, aerobic exercise, and exercise for muscle mass gain. Osteoarthritis symptoms were assessed before treatment, at the end of treatment, and 6 months posttreatment. Significant improvement in scores reflecting knee pain, stiffness, and function was seen at the end of treatment with orlistat compared with placebo (37 *versus* 21, 44.5 *versus* 28.3, and 45.5 *versus* 27.1, respectively), along with a reduction in BMI (32.9 kg/m^2^ *versus* 29.5 kg/m^2^, respectively). Although the mean BMI returned to the baseline value (31.1 kg/m^2^) after 6 months, the improvement in the other parameters persisted (23.9, p = 0.028; 27.1, p = 0.028; and 32.9, p = 0.037, respectively) (49).

#### 2.5.14 Effects of orlistat in patients with chronic kidney disease

We found no studies evaluating orlistat in chronic kidney disease (CKD).

#### 2.5.15 Effects of orlistat in patients with cardiovascular outcomes

We found no RCTs evaluating the cardiovascular safety of orlistat.

## 3. LIRAGLUTIDE

### 3.1 Mechanism of action

Liraglutide is a glucagon-like peptide-1 (GLP-1) analogue (GLP-1a) that shares 97% homology with the native GLP-1. Structural modifications to the protein increased its circulation half-life from 1-2 minutes to 13 hours (50). Liraglutide acts on hypothalamic neurons involved in energy balance and centers linked to pleasure and reward, stimulates pancreatic glucose-dependent insulin production, inhibits glucagon and somatostatin, and slows gastric emptying (51).

### 3.2 Dosage/usage instructions

Liraglutide 3.0 mg was approved by the US Food and Drug Administration (FDA) in 2014 for treating obesity; this dose was higher than the one previously approved for treating T2DM (1.8 mg). The medication should be introduced gradually to minimize side effects, which are commonly gastrointestinal in nature. Liraglutide comes with a delivery system containing 3 mL, capable of dispensing doses of 0.6 mg, 1.2 mg, 1.8 mg, 2.4 mg, or 3.0 mg. The treatment should begin with 0.6 mg/day subcutaneously and increase by 0.6 mg each week until reaching the maximum dose of 3.0 mg/day.

### 3.3 Tolerability/side effects

The most common adverse events are mainly related to the gastrointestinal system and affect more than 5% of patients. These side effects include nausea, vomiting, diarrhea, constipation, abdominal pain, and dyspepsia. In 94% of cases, these events are mild or moderate, usually related to the medication dose (hence the recommendation for gradual increase), transient, and rarely lead to treatment interruption (52).

Serious adverse events affect more than 0.2% of patients and include a higher incidence of cholelithiasis and acute cholecystitis, attributed to both weight loss and reduced gallbladder contractility. The risk of pancreatitis was slightly higher in the liraglutide group (0.4%) than the placebo group (0.1%), but this difference was not significant (52). The medication has an overall excellent safety profile, including in neuropsychiatric aspects, with no interaction with centrally acting medications, and demonstrates good efficacy.

### 3.4 Absolute contraindications

The few contraindications to liraglutide include pregnancy, breastfeeding, and hypersensitivity to the drug or its excipients. Caution is recommended when liraglutide is used by patients with a previous history of acute pancreatitis. Its use should be avoided by patients with a personal or family history of multiple endocrine neoplasia or medullary thyroid cancer, as the drug has been shown to induce thyroid C-cell hyperplasia in rodents (53).

### 3.5 Efficacy

#### 3.5.1 Effects of liraglutide on body weight

Preliminary studies have shown significantly greater weight loss with liraglutide than placebo or orlistat (54,55). Subsequently, a series of studies named Satiety and Clinical Adiposity – Liraglutide Evidence (SCALE) analyzed the use of liraglutide in the treatment of obesity and its complications. In the SCALE Obesity and Prediabetes study, 63.2% and 33.1% of the patients lost, respectively, more than 5% and 10% of their initial weight after 56 weeks (52). The study continued for another 2 years, to a total of 3 years, in patients with prediabetes. The 5%, 10%, and 15% weight loss in patients randomized to liraglutide were 49.6%, 24.8%, and 11%, respectively (56). In the SCALE Maintenance study, patients with obesity who lost 6% of weight with diet and physical activity were randomized to liraglutide 3.0 mg or placebo for 1 year. Those who used liraglutide had an additional loss of 6.1% compared with those who used placebo, reinforcing the importance of chronic and multidisciplinary treatment of obesity (57). A recent study evaluated patients who lost an average of 13.1 kg over 8 weeks on a low-calorie diet. Those who were subsequently randomized to a combination of liraglutide 3.0 mg and physical exercise achieved an additional weight loss of 3.4 kg, and at 1 year, 33% were able to maintain a weight loss of over 20% of their initial weight (58).

#### 3.5.2 Effects of liraglutide on body weight maintenance

The effects of liraglutide on weight loss maintenance were evaluated in the SCALE Maintenance study described previously (57).

#### 3.5.3 Effects of liraglutide on body composition

The study cited previously also analyzed body composition using DXA and reported a 3.9% reduction in absolute body fat percentage, which was double the decrease observed in the exercise group (1.7%) (58). Another study published in the same year assessed the use of liraglutide 3.0 mg in decreasing visceral fat, evaluated using magnetic resonance imaging. At 36 weeks, there was an average 12.5% reduction with liraglutide compared with 1.6% with placebo (59).

#### 3.5.4 Effects of liraglutide in patients with prediabetes/glucose intolerance

The effects of liraglutide in preventing the progression of prediabetes to T2DM and improving insulin resistance with weight loss are well established (59). However, studies with animal models suggest other complex direct actions of liraglutide in inhibiting the progression of prediabetes (60,61). Some clinical studies have evaluated the effects of liraglutide in individuals with prediabetes.

Kim and cols. compared the effects of liraglutide doses up to 1.8 mg *versus* placebo in a group of patients aged 40-70 years with overweight or obesity and prediabetes. Weight loss associated with liraglutide was accompanied by a 29% reduction in peripheral insulin resistance, as assessed by the insulin suppression test. Additionally, 75% of the individuals on liraglutide achieved normal fasting plasma glucose compared with 19% of those on placebo (62).

The most important RCT was the SCALE Obesity and Prediabetes trial, in which 2,254 patients with overweight or obesity and prediabetes were randomized, in a 2:1 ratio, to liraglutide 3.0 mg or placebo, combined with a standardized diet and exercise. The study showed significant and sustained results of improved glycemic control with reduced insulin resistance in the context of 6.1% weight loss over 3 years in patients using liraglutide. Only 2% of the participants in the liraglutide group developed diabetes, compared with 6% in the placebo group. Liraglutide led to an approximately 80% reduction in T2DM risk, and the estimated time to onset of T2DM over 160 weeks was 2.7 times longer in the liraglutide group compared with the placebo group. Furthermore, at 160 weeks, 66% of patients on liraglutide achieved normoglycemia, compared with 36% of those on placebo. An additional *post hoc* analysis was conducted at week 172 to address the lack of follow-up data for withdrawn participants, assuming that diabetes was undiagnosed in 1% of the participants withdrawn from the liraglutide group and in 0% of those withdrawn from the placebo group. The results showed that the risk of T2DM remained 66% lower in the participants who received liraglutide (52,56).

#### 3.5.5 Effects of liraglutide on glycemic control in patients with type 2 diabetes mellitus

Considering that controlling excess weight is one of the priorities in T2DM management, liraglutide has become one of the first-choice treatments for patients with T2DM and obesity due to its mechanism of action of direct hypoglycemic effects and body weight reduction (63).

The safety, tolerability, and efficacy of liraglutide were initially assessed in the treatment of T2DM through the Liraglutide Effect and Actions in Diabetes (LEAD) program. This program consisted of six RCTs that assessed liraglutide as a standalone treatment and in combination with oral antidiabetic drugs (OADs) at different stages of the disease. Levels of HbA1c decreased by 0.8%-1.6% from baseline with liraglutide at doses up to 1.8 mg (64). Rapid and sustained reductions in fasting plasma glucose level (up to 43.2 mg/dL) were observed from baseline to the end of each LEAD study. Liraglutide also effectively reduced postprandial glucose levels, with a mean reduction over three meals of up to 48.6 mg/dL across the six LEAD studies. These RCTs also confirmed a low risk of hypoglycemia with liraglutide, which is consistent with its glucose-dependent insulin secretion stimulating action (65).

The SCALE Diabetes study included 846 adults with overweight or obesity and with T2DM, randomized to receive liraglutide 3.0 mg, liraglutide 1.8 mg, or placebo for 56 weeks. Reductions in HbA1c level from baseline were 1.3%, 1.1%, and 0.3% in each group, respectively, and the percentages of individuals achieving HbA1c level of 6.5% or lower at the end of the study were 56.5%, 45.6%, and 15%, respectively. Liraglutide 3.0 mg was significantly superior to liraglutide 1.8 mg regarding glucose-related measures, including HbA1c values, fasting plasma glucose, fasting proinsulin, proinsulin-to-insulin ratio, and change in OAD association. However, the study authors advised caution in interpreting the comparison between the two doses, as the analyses were not controlled for multiplicity (66).

A systematic review published in 2016 included 43 studies conducted in Europe (n = 24), the United States (n = 5), and Asia-Pacific (n = 14), evaluating a total of 7,413 patients with T2DM treated with liraglutide as monotherapy or combined with hypoglycemic agents. The studies ranged in duration from 3 to 24 months (46.5%; n = 20 with ≥ 12 months) and assessed liraglutide doses between 0.9 and 1.8 mg. Liraglutide treatment resulted in HbA1c changes from -0.6% to -2.26% and reduced plasma glucose values, regardless of baseline HbA1c levels and follow-up duration. Overall, 29.3%-64.5% and 22%-41% of patients with T2DM treated with liraglutide achieved target HbA1c levels of 7% and 6.5%, respectively. Over time, treatment with liraglutide resulted in a mean change of -1.3 to -8.7 kg in absolute weight from baseline. Hypoglycemia with liraglutide monotherapy occurred at a ≤ 0.8% rate and was more frequent in patients using liraglutide combined with hypoglycemic agents (0-15.2%) (67).

A subsequent multicenter study conducted across 45 diabetes clinics in Italy included 1,723 patients who received liraglutide doses of up to 1.8 mg and were followed for up to 24 months. In all, 43.5% of the patients achieved a reduction in HbA1c of ≥ 1% in 12 months, and 40.9% reached the HbA1c target of ≤ 7% at 24 months with liraglutide monotherapy or combined with other hypoglycemic agents (68). Other studies in a “real-world” context have confirmed the glycemic control results observed in RCT conditions (69).

#### 3.5.6 Effects of liraglutide on lipid metabolism

Studies in animals and humans suggest that liraglutide may have some effects on lipid metabolism, independent of weight loss. In rats, the effects of liraglutide have been shown to impact pathways involved in increased cholesterol efflux (70) and in the expression of genes involved in the breakdown of lipoproteins containing apolipoprotein (apo) B-100, which is the main component of very-low-density lipoprotein cholesterol (VLDL-c), intermediate-density lipoprotein cholesterol (IDL-c), LDL-c, and lipoprotein (a) particles (71). In the same study, treatment of patients with T2DM with liraglutide 1.2 mg for 6 months significantly reduced plasma apo B-100 and fasting triglyceride levels and induced breakdown of triglyceride-rich lipoproteins (VLDL-c and IDL-c) and LDL-c (71).

Taskinen and cols. observed specific effects of liraglutide 1.8 mg on postprandial chylomicron metabolism in a small group of individuals with T2DM. Liraglutide led to a marked decrease in apo B-48 production in the intestine, increased the size of postprandial chylomicrons in circulation, dramatically reduced the direct clearance of chylomicrons, and decreased the hepatic secretion of VLDL-triglycerides (72). In another study, liraglutide reduced postprandial hyperlipidemia by increasing apo B-48 catabolism and reducing apo B-48 production in patients with T2DM (73).

In a Finnish study center, 22 patients with T2DM using metformin and statin were randomized to receive liraglutide 1.8 mg or placebo for 16 weeks. At the end of the study, serum concentrations of triglycerides, chylomicrons, and large VLDL-c particles after a high-fat mixed meal were significantly lower in the liraglutide group but not in the placebo group, despite similar weight losses in both two groups. Concentrations of apo C-III, a critical regulator of postprandial triglyceride metabolism, decreased markedly in the fasting and postprandial periods in the liraglutide group but not in the placebo group (74).

A meta-analysis of the results of the LEAD trials revealed significant reductions from baseline in total cholesterol (5.0 mg/dL), LDL-c (7.7 mg/dL), and triglycerides (17.7 mg/dL; p < 0.01 for all) among patients treated with liraglutide 1.8 mg, although these reductions were not significant compared with placebo or active comparators (61). In contrast, the SCALE Diabetes study showed that liraglutide 3.0 mg, but not liraglutide 1.8 mg, significantly improved total cholesterol, VLDL-c, HDL-c, and triglyceride levels compared with placebo; no effects were observed on levels of LDL-c or free fatty acids (66).

#### 3.5.7 Effects of liraglutide on blood pressure and heart rate

Studies have confirmed the effect of liraglutide on reducing BP values. This effect was attributed not only to the associated weight loss but also to a combination of other mechanisms, such as the promotion of natriuresis (75) and vasodilation (76).

Notably, GLP-1as are generally associated with a slight increase in heart rate. Current data indicate that this effect does not result in increased cardiovascular risk, although a pronounced increase in heart rate may be associated with adverse clinical outcomes in patients with advanced HF (77).

A pooled analysis of the six LEAD RCTs, including data from almost 2,800 individuals with T2DM, showed that participants receiving liraglutide experienced significantly greater mean reductions in SBP values than those receiving placebo at 26 weeks relative to baseline. These reductions were noticeable after 2 weeks of treatment. Although the trials were not statistically powered to evaluate BP reduction, consistent reductions were observed in SBP values with liraglutide (1.8 mg or 1.2 mg once daily), with reductions of 2.1-6.7 mmHg from baseline to the end of the treatment period (26-52 weeks). Small and nonsignificant reductions from baseline in DBP values were observed with liraglutide in most of these trials. The SBP reductions observed in patients treated with liraglutide correlated weakly with weight loss. Liraglutide 1.2 mg and 1.8 mg were associated with a significant mean increase of 3 beats per minute (bpm) in pulse rate, compared with a mean increase of 1 bpm with placebo (78). A similar heart rate increase with liraglutide (3 bpm) has also been found in the LEADER study, which will be detailed later (79).

Kumarathurai and cols. observed a significant increase in heart rate and reduction in heart rate variability (HRV) in patients with newly diagnosed T2DM and stable CAD who received liraglutide 1.8 mg for 12 weeks compared with placebo. This HRV reduction was not mediated by the increased heart rate observed after liraglutide therapy, suggesting a direct influence of liraglutide on sympathovagal balance (80).

In an RCT, liraglutide was associated with a significant SBP reduction compared with placebo when added to patients with T2DM already treated with multiple daily insulin injections. Although significant correlations were found between reductions in SBP and reductions in body weight and BMI, one in three liraglutide-treated patients who experienced a marked reduction in SBP did not have a substantial decrease in body weight. A greater SBP reduction was predicted by higher baseline DBP values and by lower baseline mean values of glucose regulation parameters. One explanation for this latter finding is that patients with higher mean values of glucose regulation parameters are more likely to experience blood glucose improvement with liraglutide, which decreases glycosuria and, thus, attenuates weight loss. Therefore, from a BP perspective, some patients may benefit from the use of liraglutide despite not having improvements in other traditional metabolic risk factors (81).

Zhao and cols. evaluated the effect of liraglutide on BP in a meta-analysis of 18 RCTs. The authors observed that, compared with placebo, liraglutide reduced SBP by 3.18 mmHg but had no significant effect on DBP. Only three RCTs evaluated the effect of liraglutide at the doses of 2.4 mg and 3.0 mg. Although no RCTs have been published on liraglutide 3.0 mg specifically among patients with obesity and hypertension, a subgroup analysis defined by liraglutide dose, compared with placebo, showed significant SBP reductions with the doses of 2.4 mg/day (-5.01 mmHg) and 3.0 mg/day (-3.67 mmHg) and DBP reduction (-1.46 mmHg) with the dose of 3.0 mg/day (82).

#### 3.5.8 Effects of liraglutide on obstructive sleep apnea syndrome

Although the association of OSAS with both obesity and T2DM is well established (83), only a few studies have directly measured with polysomnography the effects of liraglutide in patients with OSAS.

The classic RCT SCALE Sleep Apnea evaluated the effects of liraglutide 3.0 mg in individuals with obesity and moderate or severe OSAS who were reluctant or unable to use CPAP therapy. After 32 weeks of treatment, a significantly greater reduction in mean AHI was observed in the treated group compared with the placebo group, both of which were also addressed with monthly counseling on diet and exercise (-12.2 ± 1.8 events/h *versus* -6.1 ± 2.0 events/h, respectively). The improvement in OSAS outcomes was associated with the degree of weight loss at the end of the study (84).

A recently published study included individuals with T2DM and moderate or severe OSAS randomized to a control group or a liraglutide group. Both groups used CPAP and received drug treatment for T2DM, except for the first group, which received liraglutide at a dose of up to 1.8 mg. After 3 months of follow-up, the mean BMI, AHI, and SBP values in the liraglutide group were lower than those in the control group, while minimum oxygen saturation was higher in the liraglutide group (85).

#### 3.5.9 Effects of liraglutide in patients with polycystic ovary syndrome

The effects of liraglutide in women with PCOS were assessed in a series of studies, both as a standalone and in combination with metformin, demonstrating significant weight loss and reduction in testosterone levels. The results were heterogeneous regarding insulin resistance and menstrual patterns. Most studies used liraglutide doses between 1.2 mg and 1.8 mg. Although few studies have evaluated fertility and gestational outcomes with GLP-1as, weight loss is known to be the most significant factor affecting the improvement of these parameters in PCOS (86). It is important to note that the liraglutide package insert recommends discontinuing the medication if the patient desires to become pregnant.

The effects of GLP-1as in women with PCOS have been evaluated in a meta-analysis of six studies with liraglutide (1.2-1.8 mg) and one with exenatide. A significant weight loss and reduction in total testosterone levels was observed, but no effects were found in abdominal circumference, fasting insulinemia, homeostasis model assessment of insulin resistance (HOMA-IR) values, or SHBG level. Only one study evaluated hirsutism and menstrual cycles, and this study found no significant changes after liraglutide treatment (87).

A recent meta-analysis compared the effects of liraglutide (1.2-1.8 mg), metformin, and the combination of metformin + liraglutide in women with overweight or obesity and PCOS. Compared with the group treated with metformin alone, the metformin + liraglutide group showed greater weight loss and reduction in waist circumference, fasting blood glucose, and insulin levels, but no difference in HOMA-IR values. When the standalone treatments with metformin *versus* liraglutide were compared, liraglutide was only superior to metformin in terms of weight loss. There was no significant difference between metformin, liraglutide, and combined metformin plus liraglutide in improving total testosterone, free testosterone, or SHBG levels. Although two studies reported improvements in menstrual cycles with the combined therapy compared with metformin alone, they used different indicators, hindering a meta-analysis of these data (88).

The effects of liraglutide 1.8 mg on ovarian morphology, hormonal levels, and menstrual bleeding patterns were evaluated in a double-blind RCT including 72 women with overweight or obesity and PCOS. The group treated with liraglutide experienced a reduction in ovarian volume, along with an increase in SHBG level, reduction in free testosterone level, and improvement in bleeding rate (89). While most studies evaluated lower doses of liraglutide, a double-blind RCT assessed the effects of liraglutide 3.0 mg for 32 weeks in 82 women with obesity and PCOS, reporting significant weight loss, improvement in hyperandrogenism, and restoration of menstrual cycles (90).

The pregnancy rates after *in vitro* fertilization were investigated in an open-label RCT including 28 women with obesity and PCOS, comparing the effects of metformin plus liraglutide 1.2 mg *versus* metformin alone for 12 weeks. The pregnancy rate per embryo transfer was significantly greater in the combined treatment group (85.7%) compared with the metformin alone group (28.6%), and the cumulative pregnancy rates over a 12-month period were 69.2% and 35.7%, respectively (91).

#### 3.5.10 Effects of liraglutide in patients with male hypogonadism

Studies evaluating the effects of liraglutide in patients with male hypogonadism do not allow for definitive conclusions but suggest an improvement in testosterone levels and sexual function accompanying weight loss and improvement in metabolic parameters. It is unclear whether the effects of liraglutide in patients with male hypogonadism are mediated exclusively by the reduction in adiposity. In animal models, there is evidence of direct effects of central GLP-1 signaling on the gonadal axis. Intracerebroventricular GLP-1 injection induces an immediate luteinizing hormone (LH) surge in male rats (92).

A retrospective observational study has evaluated the effects of liraglutide added to testosterone replacement therapy (TRT), metformin, and LSCs on erectile function in men with obesity, T2DM, and hypogonadism. In the first year, all 43 patients (aged 45-59 years) received TRT, metformin, and LSC recommendations. In the second year, those who did not reach the target HbA1c value received additional liraglutide 1.2 mg daily. The group that received liraglutide showed additional weight loss and improvement in erectile function compared with the group that did not receive it (93).

A prospective, randomized, open-label study evaluated the effects of liraglutide 3.0 mg daily compared with testosterone 50 mg (1% transdermal gel) for 16 weeks in 30 men with a mean age of 46 years, obesity, and functional hypogonadism. The weight loss was only significant in the group that received liraglutide. Both groups experienced improvements in total testosterone levels, libido, and sexual function. Follicle-stimulating hormone (FSH) and LH levels increased in the liraglutide group and decreased in the testosterone group (94).

In a prospective study, 110 young (aged 18-35 years) men with obesity and functional hypogonadism were divided according to their desire for fertility into three groups to receive gonadotropins, liraglutide 3.0 mg, or transdermal testosterone 60 mg for 4 months. The group that received liraglutide showed significant weight loss and higher levels of testosterone and gonadotropins, as well as improved erectile function and conventional sperm parameters relative to baseline levels and compared with the other groups (95).

#### 3.5.11 Effects of liraglutide on metabolic dysfunction-associated steatotic liver disease

Liraglutide has demonstrated benefits in patients with MASLD, reducing liver fat content and improving steatohepatitis. In addition to its weight loss effect in reducing lipotoxicity, other mechanisms have been proposed, such as modification of portal and peripheral insulin and glucagon concentrations, and improvements in hepatocyte mitochondrial function and hepatic insulin sensitivity (96).

Four RCTs showed a reduction in liver fat content assessed by magnetic resonance imaging-based techniques after treatment with liraglutide 1.8 mg for 6 months. The study evaluated adults with overweight or obesity and T2DM (97-99) and women with overweight and PCOS (100). Other studies have reported similar results.

The LEAN study, a double-blind RCT, examined the effects of liraglutide on steatohepatitis and fibrosis. Armstrong and cols. randomized 52 overweight patients with biopsy-proven steatohepatitis to receive liraglutide 1.8 mg or placebo for 48 weeks. The primary outcome of resolution of steatohepatitis occurred in 39% of patients in the liraglutide group *versus* 9% of those in the placebo group (p = 0.019), and progression of fibrosis occurred in 9% of patients receiving liraglutide and in 36% of those receiving placebo (101).

#### 3.5.12 Effects of liraglutide on quality of life

Treatment with liraglutide resulted in improved quality-of-life parameters compared with placebo in an RCT. The benefits appeared to be associated with weight loss, as they were greater in individuals with greater weight loss, regardless of treatment arm.

One of the secondary outcomes of the SCALE Obesity and Prediabetes study was health-related quality of life, assessed using the SF-36, IWQOL-Lite, and Treatment Related Impact Measure – Weight after 56 weeks of treatment with liraglutide 3.0 mg. Compared with the placebo group, the liraglutide group had higher SF-36 scores in the general physical and mental health domains, higher IWQOL-Lite total scores, and more favorable individual domain scores on both instruments. In the assessment with the Treatment Related Impact Measure – Weight, the total score was also higher in the liraglutide group, despite a lower score for the experience of side effects (102). The greatest benefits were observed in the physical aspects of the IWQOL-Lite and in self-esteem (103). Quality of life was also assessed in the continuation of the SCALE Obesity and Prediabetes study for 160 weeks, which showed that the improvement demonstrated after 1 year of treatment with liraglutide 3.0 mg was generally maintained after 3 years (104).

#### 3.5.13 Effects of liraglutide on osteoarticular diseases

While preclinical studies have suggested positive effects of GLP-1 receptor agonists in osteoarthritis (105), including direct effects on various joint cell types (106), an RCT found no significant benefits of liraglutide 3.0 mg for pain associated with knee osteoarthritis, despite a relatively small weight difference between the groups (107). Gudbergsen and cols. randomized 156 patients with overweight or obesity and knee osteoarthritis who had lost more than 5% of weight with dietary intervention for 8 weeks to receive liraglutide 3.0 mg or placebo for 52 weeks. At the end of the study, the difference in weight between the groups was 3.9 kg, and there was no significant difference in knee pain, as measured by a subscale of the Knee Injury and Osteoarthritis Outcome Score (KOOS). However, it is important to highlight that the average weight loss with the pre-randomization dietary intervention was 12.5 kg and there was a significant improvement in symptoms during this period. Consequently, at the time of randomization, the patients had mild-to-moderate pain, which may have limited the potential of pharmacologic intervention to promote further improvement (107).

#### 3.5.14 Effects of liraglutide in patients with chronic kidney disease

Kidney disease is one of the most important complications of T2DM and the most common cause of CKD and end-stage renal disease (ESRD). A recent review looked at medications with established evidence for treating diabetic kidney disease. Among them are incretin-based therapeutic agents, including liraglutide, which have demonstrated vasotropic actions, suggesting a potential to reduce the risk of diabetic kidney disease (108).

In the Liraglutide and Cardiovascular Outcomes in Type 2 Diabetes (LEADER) study, liraglutide showed cardiovascular and renal benefits, particularly in participants with CKD. The results suggested that reductions in HbA1c and SBP values may moderately mediate the renal benefits of liraglutide. Potential benefits may be driven by other mediators or direct mechanisms (109). A *post hoc* analysis evaluated the safety of liraglutide treatment in patients with CKD. A total of 9,340 patients with T2DM were randomized to receive either liraglutide or placebo, both in addition to standard treatment. Of these, 2,158 had CKD and 7,182 had no CKD (defined as an estimated glomerular filtration rate [eGFR] < 60 and ≥ 60 mL/min, respectively); 966 patients had macroalbuminuria, and 2,456 had microalbuminuria (urine albumin-to-creatinine ratio > 300 mg/g and ≥ 30 to ≤ 300 mg/g, respectively). At the beginning of the study, the mean eGFR was 46 ± 11 mL/min in patients with CKD and 91 ± 22 mL/min in those without CKD. The risk of severe hypoglycemia was significantly lower with liraglutide compared with placebo in patients with CKD or with micro- or macroalbuminuria (hazard ratio [HR] = 0.63 and 0.57, respectively). The study concluded that the use of liraglutide in patients with CKD was safe, with no difference between patients with and without CKD (110). No dosage adjustment is necessary for patients with mild or moderate renal impairment.

Experience is limited in patients with severe renal insufficiency. A study evaluated the safety and efficacy of liraglutide in patients with T2DM and ESRD dependent on dialysis. Twenty-four patients with T2DM and ESRD and 23 control individuals with T2DM and normal renal function were randomized to receive 12 weeks of liraglutide (titrated to a maximum dose of 1.8 mg) or placebo as an add-on to ongoing antidiabetic treatment. Glycemic control improved in both groups treated with liraglutide, and the basal insulin dose decreased accordingly. Body weight also decreased in both groups treated with liraglutide. The plasma concentration of liraglutide was 49% higher in the ESRD group compared with the control group. Nausea and vomiting occurred more frequently among liraglutide-treated patients with ESRD compared with control individuals. The study concluded that reduced treatment doses and a prolonged titration period may be advisable (111), although liraglutide is currently not recommended in this population.

#### 3.5.15 Effects of liraglutide on cardiovascular outcomes

Studies in animal models have shown liraglutide effects in reducing oxidative stress and inflammation and preventing apoptosis of endothelial cells; these effects were independent of glycemic control or weight loss and may contribute to the cardiovascular protective action of this drug (112,113). Beneficial effects in reducing inflammatory markers and neutralizing oxidative stress and endothelial dysfunction in individuals treated with liraglutide have also been described (112).

Cardiovascular outcomes of liraglutide were investigated in the LEADER study, in which 9,340 individuals with T2DM and high cardiovascular risk were randomized and followed for a median of 3.8 years. The group treated with liraglutide at a dose of up to 1.8 mg had a 13% reduction in primary outcomes (cardiovascular death, nonfatal AMI, or nonfatal stroke) compared with the placebo group. Mortality from cardiovascular causes was lower in the liraglutide group (4.7% *versus* 6.0% in the placebo group). Nonfatal myocardial infarction, nonfatal stroke, and hospitalizations due to HF were less frequent in the liraglutide group, but the differences compared with placebo were not significant (79).

A *post hoc* analysis of the LEADER study was performed to evaluate the treatment effect of liraglutide *versus* placebo on cardiovascular outcomes by LDL-c level < 50 mg/dL, 50-70 mg/dL, and > 70 mg/dL and statin use at the beginning of the study. The results suggest that the benefits of liraglutide on mortality and cardiovascular outcomes appear consistent in patients with T2DM at high cardiovascular risk, independent of LDL-c level, and persist even in the setting of very low baseline LDL-c levels and concomitant statin use. These data suggest that the potential antiatherosclerotic effects of the medication are complementary to its effect in reducing lipids (113).

No RCTs have been conducted to assess the cardiovascular benefits of the 3.0 mg dose in patients with obesity without T2DM. However, a *post hoc* analysis was performed using pooled data from 5,908 participants from the five RCTs of the SCALE program (liraglutide *versus* placebo or orlistat). In that study, liraglutide 3.0 mg was not associated with increased cardiovascular risk. Since wide confidence intervals were found, and two retrospective studies were included in the analysis, it is not possible to claim cardiovascular protection with the medication, only noninferiority compared with placebo regarding this outcome (114).

## 4. SEMAGLUTIDE

### 4.1 Mechanism of action

Semaglutide is a long-acting GLP-1a that mimics the effects of native GLP-1. Like other GLP-1as, semaglutide has effects in various locations and multiple actions, including reduced caloric intake, increased satiety, and decreased hunger, leading to weight loss (115). In animal models, GLP-1as act both on the hypothalamus, stimulating anorexigenic pathways, and on the mesolimbic system, influencing the reward system (116).

In a study including 72 adults with overweight or obesity and comparing semaglutide 2.4 mg *versus* placebo, *ad libitum* energy intake was 35% lower with semaglutide than placebo (1,736 *versus* 2,676 kJ, respectively; estimated treatment difference -940 kJ). Semaglutide reduced hunger and potential food intake, and increased fullness and satiety compared with placebo. The CoEQ scores indicated better dietary control and reduced food cravings with semaglutide compared with placebo (p < 0.05). These effects resulted in a 9.9% reduction in body weight with semaglutide and 0.4% with placebo (117).

Semaglutide is 89% bioavailable after subcutaneous injection. Peak concentrations occur 3 days after injection, and a steady state is reached by week 5 when injected once weekly. Similar exposure was achieved in three subcutaneous administration sites: abdomen, thigh, and upper arm. More than 99% of semaglutide binds to plasma albumin, providing protection against degradation and renal clearance. Semaglutide is modified through the substitution of alanine at position 8 to protect it from natural degradation by dipeptidyl peptidase 4 (DPP-4). The elimination half-life of semaglutide is approximately 1 week; therefore, it remains in circulation for approximately 5-7 weeks after the last dose. Semaglutide is eliminated in urine and feces. No dosage adjustments are required based on hepatic or renal function (115,118).

### 4.2 Dosage/usage instructions

Weight loss with semaglutide is dose-dependent, with higher doses resulting in greater weight loss. The package insert recommends an initial subcutaneous dose of 0.25 mg once weekly, with no relation to meal times. The dose should be titrated every 4 weeks, increasing to 0.5 mg, 1.0 mg, 1.7 mg, and 2.4 mg, which is the maximum effective dose for weight loss. In patients with poor tolerance to dose titration, it is recommended to consider a 4-week “delay” in dose titration, *i.e.*, to maintain the maximum tolerated dose for 4 weeks longer before attempting a new dose increase. The goal should be toward the maximum tolerated dose, although some patients in clinical practice are “hyper-responders” and experience significant weight loss with lower doses (118).

### 4.3 Tolerability/side effects

The most common side effects of semaglutide occur in the gastrointestinal tract, as with other GLP-1as.

The Semaglutide Treatment Effect in People with Obesity (STEP) studies were a pivotal phase 3 clinical trial series evaluating subcutaneous semaglutide 2.4 mg weekly for weight loss. Data from these studies served for discussions regarding the weight loss efficacy, safety profile, tolerability, and effects of semaglutide on cardiometabolic parameters. In STEP 1, nausea, diarrhea, vomiting, and constipation occurred in 74.2% of participants in the semaglutide group and 47.9% of those in the placebo group. As a rule, events were mild to moderate in severity and transient, resolving permanently after treatment discontinuation. Gallbladder-related disorders (mainly cholelithiasis) were reported in 2.6% and 1.2% of participants in the semaglutide and placebo groups, respectively. Three participants in the semaglutide group had mild acute pancreatitis (two had gallstones); all participants made a full recovery. Serious adverse events were reported in 9.8% of patients in the semaglutide group and 6.4% of those in the placebo group and included mainly severe gastrointestinal and hepatobiliary events. One death was reported in each group, and neither was considered related to the receipt of semaglutide or placebo, as assessed by an independent external event adjudication committee. There was no difference between groups regarding the incidence of benign or malignant neoplasms, cardiovascular events, acute renal failure, or hypoglycemia (119).

The other STEP studies had a similar pattern of side effects. In a meta-analysis of four studies, including three studies from the STEP series and with a total of 3,613 patients, Tan and cols. found that the risk of gastrointestinal adverse events was 1.59 times higher with semaglutide (RR = 1.59, 95% CI = 1.34-1.88). The risk of discontinuation due to adverse events was twice as high in the semaglutide group (RR = 2.19), and the risk of severe adverse events (SAEs), particularly biliary tract diseases (cholelithiasis and cholecystitis) and acute pancreatitis, was 1.6 times higher in the semaglutide group (120).

In a large database analysis conducted in the United States (PharMetrics Plus) with approximately 16 million patients, Sodhi and cols. compared users of the GLP-1as liraglutide and semaglutide with those using the combination of naltrexone and bupropion (N/B). They found that GLP-1a use was associated with an increased risk of pancreatitis (HR = 9.09), intestinal obstruction (HR = 4.22), and gastroparesis (HR = 3.67), but not of biliary disease (HR = 1.50, nonsignificant), differing from findings in the STEP studies. Two aspects of the study must be highlighted: first, the confidence interval was very wide, suggesting that the sample was not adequate; second, the indiscriminate use of GLP-1as may have increased the risk of side effects, among other consequences (121).

### 4.4 Absolute contraindications

The use of semaglutide is contraindicated during pregnancy and in cases of hypersensitivity to semaglutide or any of its excipients (122).

### 4.5 Efficacy

#### 4.5.1 Effects of semaglutide on body weight

The efficacy of semaglutide for weight loss was initially demonstrated in a phase 2 study, in which patients with overweight or obesity and without T2DM were divided into seven groups: five using daily subcutaneous semaglutide at different doses (0.05 mg, 0.1 mg, 0.2 mg, 0.3 mg, and 0.4 mg), one using liraglutide 3.0 mg, and one using placebo. At the end of the study, the mean weight loss in patients using semaglutide was 6.0% (0.05 mg), 8.6% (0.1 mg), 11.6% (0.2 mg), 11.2% (0.3 mg), and 13.8% (0.4 mg), showing a clear superiority of semaglutide over placebo (2.3%). Starting at a daily dose of 0.2 mg, which is equivalent to 1.4 mg per week, weight loss with semaglutide was greater than that with liraglutide (7.8%) (123).

In STEP 1, a total of 1,961 patients with overweight or obesity and without T2DM were evaluated and followed up for 68 weeks. All participants were instructed to follow a hypocaloric diet with a 500 kcal/day deficit and practice 150 minutes of physical activity per week. At the end of the study, participants in the semaglutide 2.4 mg group lost on average 16.9% of weight, while those in the placebo group lost 2.4%. The nadir was reached at week 60 (119).

The STEP 2 study evaluated 1,210 patients with T2DM with a BMI > 27 kg/m^2^ and HbA1c levels between 7.0% and 10%. The patients were divided into three groups: semaglutide 2.4 mg, semaglutide 1.0 mg, and placebo. At 68 weeks, semaglutide 2.4 mg led to greater weight loss than semaglutide 1.0 mg. Patients in the semaglutide 2.4 mg group lost an average of 9.6% of their body weight, compared with 7.0% in the semaglutide 1.0 mg group and 3.4% in the placebo group (124). A comparison of the results of the STEP 1 and STEP 2 studies showed that the participants with T2DM lost less weight than those without T2DM, replicating the finding by studies conducted with other medications.

The STEP 3 study evaluated 611 patients and had a design virtually identical to that of STEP 1, differing only in the degree of LSCs, which were more intensive. At the end of the 68-week period, the intervention group lost an average of 16% of body weight, while the placebo group lost 5.7% (125).

The STEP 4 study was designed to assess the effects of continuing *versus* interrupting semaglutide treatment in individuals with overweight or obesity. A total of 902 patients received semaglutide in escalating doses of up to 2.4 mg/week, with an average weight loss of 10%. At week 20, half of the group was randomized to continue on semaglutide while the other half was switched to placebo. At the end of the study, at week 68, the semaglutide group had an additional weight loss of approximately 7.9%, with an average weight loss of 17.4%, while the group that interrupted treatment had an average weight regain of 6.9%, with an average weight loss of 5.0%. The results of this study highlighted the importance of maintaining pharmacologic treatment in patients with obesity (126).

The STEP 5 study was designed to evaluate the long-term effects of subcutaneous semaglutide 2.4 mg once weekly compared with placebo, as an add-on to behavioral intervention, on body weight and cardiometabolic risk factors in adults with overweight or obesity. At follow-up week 104, the mean decrease in body weight was 15.2% in the semaglutide group and 2.6% in the placebo group, demonstrating the long-term efficacy of the treatment (127).

A widely used way of assessing weight loss is by evaluating weight loss categories, *i.e.*, classifying weight loss into different categories, generally based on the percentage of weight loss. Table 1 highlights the categorical weight loss observed in the STEP series studies.

**Table 1:** Categorical weight loss with weekly semaglutide 2.4 mg in the STEP series studies, divided according to the percentage of weight lost

Study/duration	Population	Loss > 5%	Loss > 10%	Loss > 15%	Loss > 20%
STEP 1 (68 weeks)	Individuals with overweight or obesity and without T2DM	86.4%	69.1%	50.5%	34.8%
STEP 2 (68 weeks)	Individuals with T2DM, with BMI > 27 kg/m^2^ and HbA1c between 7.0% and 10%	73.2%	49.9%	28.2%	14.2%
STEP 3 (68 weeks)	Same as STEP 1, with more intense lifestyle changes	86.6%	75.3%	55.8%	35.7%
STEP 4 (68 weeks)	Same as STEP 1, but evaluated the effects of interrupting *versus* continuing medication (weight maintenance)	88.7%	79.0%	63.7%	39.6%
STEP 5 (104 weeks)	Same as STEP 1, but longer study duration	77.1%	61.8%	52.1%	36.1%

Prepared by the authors.

#### 4.5.2 Effects of semaglutide on weight maintenance

See the description of the STEP 4 study above.

#### 4.5.3 Effects of semaglutide on body composition

Modification of body composition is an increasingly valued parameter in studies with antiobesity drugs. The therapeutic target is quality weight loss, *i.e.*, weight loss at the expense of fat mass with preservation or minimal loss of lean mass. The effects of semaglutide on body composition were investigated in the STEP 1 study, where a subgroup of 140 participants underwent body composition analysis using DXA. Despite a decrease in lean mass in absolute terms (-5.26 kg in the semaglutide group *versus* 1.83 kg in the placebo group; difference -3.43 kg), there was a predominant reduction in fat mass (-8.36 kg in the semaglutide group *versus* -1.37 kg in the placebo group; difference -6.99 kg), which resulted in patients having decreased percentage of body fat at the end of the study (119).

#### 4.5.4 Effects of semaglutide in patients with prediabetes/glucose intolerance

In a *post hoc* analysis of the STEP 1, 3, and 4 studies, including approximately 3,375 patients with overweight or obesity and prediabetes, the intervention group (semaglutide 2.4 mg) experienced improvement in all glycemic parameters after 68 weeks of treatment, with reductions in the risk of progression from prediabetes to T2DM between 84% and 89%, demonstrating the therapeutic potential of the drug (128).

The STEP 10 study evaluated the effects of semaglutide 2.4 mg on reversing prediabetes to normoglycemia in patients with obesity. A total of 207 patients were randomized, including 138 to the semaglutide group and 69 to the placebo group. At 52 weeks, 81.1% of patients treated with semaglutide showed blood glucose normalization compared with 14.1% of those treated with placebo (OR = 19.8; p < 0.0001). Regarding HbA1c, the average level at baseline was 5.9% and at week 52, the level was 0.5% lower in the semaglutide group compared with the placebo group (129).

#### 4.5.5 Effects of semaglutide in patients with type 2 diabetes mellitus

The efficacy of semaglutide for glycemic control was well demonstrated in the SUSTAIN series of studies, where semaglutide was administered subcutaneously at a dose of 1.0 mg/week, and in the PIONNER series of studies, where it was administered orally at a dose of up to 14 mg/day (130). These two development programs included only patients with T2DM and will not be discussed in this document.

In the STEP 2 study, the 2.4 mg dose was tested in overweight patients with T2DM. At the end of the 68-week follow-up period, the HbA1c level decreased by 1.6%, which was not significantly different from the 1.5% decrease with the 1.0 mg dose (124). In a meta-analysis assessing changes in cardiometabolic parameters, semaglutide treatment of patients with overweight or obesity without T2DM resulted in a 7.5% reduction in fasting blood glucose.

#### 4.5.6 Effects of semaglutide on lipid profile

A meta-analysis evaluating changes in cardiometabolic parameters in patients with overweight or obesity and without T2DM found that semaglutide reduced serum levels of LDL-c by 6%, triglycerides by 18%, and non-HDL-c by 8%, but did not change significantly the HDL-c level (131).

#### 4.5.7 Effects of semaglutide on blood pressure and heart rate

In a meta-analysis including 4,744 patients, semaglutide resulted in mean decreases of 4.83 mmHg in SBP and 2.45 mmHg in DBP among patients with obesity without T2DM. All GLP-1as increase heart rate, and this applies to semaglutide as well. Semaglutide leads to an average heart rate increase of 2-5 bpm. However, this effect appears to be caused by direct stimulation of the sinus node rather than reflex tachycardia due to stimulation of the autonomic nervous system and is not associated with an increased risk of adverse cardiac events (132).

#### 4.5.8 Effects of semaglutide on polycystic ovary syndrome

Jensterle and cols. randomized 25 women with obesity and PCOS (33.7 ± 5.3 years, BMI 36.1 ± 3.9 kg/m^2^) to receive semaglutide 1.0 mg or placebo for 16 weeks. The authors assessed the participants’ tongues in regard to volume, fat tissue, and fat proportion using magnetic resonance imaging. Tongue fat tissue and fat proportion reduced significantly after semaglutide *versus* placebo (-1.94 ± 5.51 cm^3^*versus* 3.12 ± 4.87 cm^3^ and 0.02 ± 0.07 cm^3^*versus* 0.04 ± 0.06 cm^3^, respectively). Correlation analysis revealed that these reductions were associated with those in body weight, BMI, and waist circumference (133). This was the first study confirming the beneficial effect of semaglutide among women with obesity and PCOS.

Recommendations on PCOS were recently published by a global task force (Recommendations from the 2023 International Evidence-based Guideline for the Assessment and Management of Polycystic Ovary Syndrome). In the absence of adequate evidence, the consensus recommendations were prepared by the committee in collaboration with consumer organizations. Recommendation 4.5.1 states that “antiobesity medications, including liraglutide, semaglutide, both glucagon-like peptide-1 (GLP-1) receptor agonists and orlistat, could be considered, in addition to active lifestyle intervention, for the management of higher weight in adults with PCOS as per general population guidelines” (134).

#### 4.5.9 Effects of semaglutide on obstructive sleep apnea syndrome

No studies specifically on semaglutide and OSAS are currently available.

#### 4.5.10 Effects of semaglutide in patients with male hypogonadism

No studies specifically on semaglutide and male hypogonadism are currently available.

#### 4.5.11 Effects of semaglutide on metabolic dysfunction-associated steatotic liver disease

A phase 2 RCT included 320 patients with biopsy-confirmed NASH and liver fibrosis who were randomized to receive subcutaneous semaglutide at daily doses of 0.1 mg, 0.2 mg, or 0.4 mg or placebo for 72 weeks. The primary endpoint of NASH resolution without worsening fibrosis was achieved by 40%, 36%, and 59% of participants in the semaglutide 0.1 mg, 0.2 mg, and 0.4 mg groups, respectively, compared with 17% of those in the placebo group (p < 0.001). However, no difference between the groups was observed regarding improvement in fibrosis stage. In conclusion, semaglutide treatment of patients with NASH and fibrosis led to a significantly higher number of patients experiencing resolution of NASH compared with placebo treatment, with no difference in improvement in fibrosis stage (135).

Another phase 2, double-blind, placebo-controlled study included 71 patients with biopsy-confirmed NASH-related cirrhosis and BMI ≥ 27 kg/m^2^. In all, 49 (69%) patients were of the female sex. The patients had a mean age of 59.5 years and a mean BMI of 34.9 kg/m^2^; 53 (75%) patients had T2DM. In total, 47 patients were randomized to the semaglutide group and 24 patients to the placebo group. After 48 weeks, there was no significant difference between the two groups regarding the proportion of patients with improvement in liver fibrosis of one stage or more without worsening of MASLD (5 [11%] of 47 patients in the semaglutide group *versus* 7 [29%] of 24 patients in the placebo group; HR = 0.28; p = 0.087). There was also no significant difference between groups in the proportion of patients achieving NASH resolution (p = 0.29). Similar proportions of patients in each group reported adverse events (42 [89%] patients in the semaglutide group *versus* 19 [79%] patients in the placebo group) and SAEs (6 [13%] patients *versus* 2 [8%] patients, respectively). The most frequent adverse events were nausea (21 [45%] *versus* 4 [17%]), diarrhea (9 [19%] *versus* 2 [8%]), and vomiting (8 [17%] *versus* none). Liver and kidney functions remained stable. There were no events of hepatic decompensation or deaths. In conclusion, semaglutide did not significantly improve fibrosis or lead to NASH resolution compared with placebo among patients with NASH and compensated cirrhosis (136). An ongoing phase 3 study of semaglutide in individuals with MASLD/metabolic dysfunction-associated steatohepatitis (MASH) is scheduled to be completed in 2028.

#### 4.5.12 Effects of semaglutide on quality of life

The quality of life of patients participating in clinical studies can be assessed using quality of life scores. In studies with semaglutide, the questionnaires used for this purpose were the SF-36 and IWQOL-Lite-CT. In the STEP studies, a significant improvement in quality of life was observed among patients using semaglutide when compared with placebo (123-127).

#### 4.5.13 Effects of semaglutide on osteoarticular diseases

The STEP 9 RCT included individuals with obesity and a clinical diagnosis of knee osteoarthritis with radiological findings and pain (Western Ontario and McMaster Universities Osteoarthritis Index [WOMAC] pain subscale score ≥ 40). The individuals were randomized to semaglutide 2.4 mg (n = 271) or placebo (n = 136) and were followed for 68 weeks. In addition to weight loss, patients randomized to semaglutide experienced significantly greater reduction in the pain scale (-41.7 points) compared with those randomized to placebo (-25.5 points; difference -14.1 points; p < 0.001), along with improvement in the subscale assessing physical function and reduced use of analgesics (137).

#### 4.5.14 Effects of semaglutide in patients with chronic kidney disease

In a real-world study of 122 patients with obesity and T2DM, treatment with semaglutide resulted in weight loss, reduced blood glucose levels, and a 50% decrease in albuminuria, with no impact on eGFR. The treatment withdrawal rate due to side effects was 5.9%, which is similar to that observed in studies carried out with patients without CKD (138). In a *post hoc* analysis of the STEP 1, 3, and 4 studies, the use of semaglutide also decreased albuminuria in patients with overweight or obesity and without diabetes, with no effects on eGFR (139).

A prespecified analysis of the SELECT study (described in item 4.5.15, “Effects of semaglutide on cardiovascular risk protection”) evaluated the effects of semaglutide on renal outcomes. The outcomes assessed included death from renal causes, initiation of dialysis therapy or renal transplantation, development of eGFR < 15 mL/min/1.73 m^2^, persistent reduction of over 50% in eGFR compared with baseline, and development of persistent macroalbuminuria. Patients randomized to semaglutide had a 22% reduction in this composite outcome (HR = 0.78; p = 0.02), with the endpoints determined primarily by persistent ≥ 50% reduction of eGFR and the onset of macroalbuminuria. Treatment with semaglutide also led to a smaller absolute reduction in eGFR compared with placebo (-0.86 mL/min/1.73 m^2^ *versus* -1.61 mL/min/1.73 m^2^, respectively) after 104 weeks and had an effect on reducing albuminuria (140).

Finally, the results of the FLOW study, which evaluated the effects of semaglutide 1.0 mg in patients with T2DM and CKD, have been published. The outcomes were similar to those previously described in the SELECT study, with cardiovascular death also included as a primary outcome. The study was interrupted prematurely due to efficacy, with the semaglutide 1.0 mg group demonstrating a 24% reduction in the primary outcome (141).

#### 4.5.15 Effects of semaglutide on cardiovascular risk protection

The cardiovascular safety of semaglutide was investigated in patients with T2DM and high cardiovascular risk in the SUSTAIN-6 study, with 3,297 patients randomized to weekly subcutaneous semaglutide 0.5 mg or 1.0 mg or placebo, for 104 weeks. The primary composite outcome of cardiovascular death, nonfatal AMI, or nonfatal stroke occurred in 108 of 1,648 patients (6.6%) in the semaglutide group and in 146 of 1,649 patients (8.9%) in the placebo group (odds ratio [OR] = 0.74; 95% CI, 0.58-0.95; p < 0.001 for noninferiority) (142).

The results of the SELECT study, the first study to demonstrate the cardiovascular benefit of a medication in individuals with obesity without diabetes, were published in 2023. In this multicenter, double-blind RCT designed to assess superiority, more than 17,000 patients with BMI ≥ 27 kg/m^2^ and CVD were randomized to receive weekly 2.4 mg of subcutaneous semaglutide or placebo. During a median follow-up of 39.8 months, a primary event (cardiovascular death, nonfatal AMI, or nonfatal stroke) occurred in 569 of 8,803 patients (6.5%) in the semaglutide group and in 701 of 8,801 patients (8.0%) in the placebo group (OR = 0.80; 95% CI, 0.72-0.90; p < 0.001). The study concluded that semaglutide 2.4 mg was superior to placebo, leading to a 20% reduction in the incidence of cardiovascular events in patients with overweight or obesity and established CVD (143).

Another landmark study was the STEP-HFpEF, the first study evaluating the effects of a GLP-1a in patients with HF with preserved ejection fraction (HFpEF). The RCT evaluated the impact of 52 weeks of treatment with semaglutide 2.4 mg in 529 patients with HFpEF and obesity. The primary outcomes were symptom improvement (assessed using the Kansas City Cardiomyopathy Questionnaire Clinical Summary Score [KCCQ-CSS]) and body weight reduction. Secondary outcomes included changes in the 6-minute walk distance and reductions in high-sensitivity C-reactive protein (CRP), among others. Patients randomized to semaglutide had a significant reduction in KCCQ-CSS scores relative to placebo (-16.6 points *versus* -8.7 points, respectively), significant body weight loss (-13.3% *versus* -2.6%, respectively), and reduction in secondary outcomes (including a reduction in high-sensitivity CRP). In patients with obesity and HFpEF, treatment with semaglutide led to improvement in symptoms, physical limitations, and exercise capacity (144).

## 5. NALTREXONE AND BUPROPION

### 5.1 Mechanism of action

Bupropion is a dopamine and norepinephrine reuptake inhibitor recommended for the treatment of depression and smoking cessation. It has an anorectic effect, related to the stimulation of pro-opiomelanocortin (POMC) neurons located in the arcuate nucleus (ARC) of the hypothalamus. These neurons release alpha-melanocyte-stimulating hormone (α-MSH), which acts on MC4R, decreasing food intake and increasing energy expenditure. Despite the demonstration of this effect in animal models, clinical studies have shown a modest weight-reducing effect of bupropion monotherapy, causing it not to meet the criteria for approval as a monotherapy for obesity (145).

Naltrexone is an opioid receptor antagonist used primarily in the treatment of alcohol and opioid dependence. It is metabolized by the hepatic enzyme dihydrodiol dehydrogenase into its active metabolite 6β-naltrexol. Both naltrexone and 6β-naltrexol are competitive antagonists at μ- and κ-opioid receptors in the central nervous system (CNS). In POMC neurons, β-endorphin release exerts negative feedback by binding to μ-opioid receptors on the POMC neuron itself, decreasing α-MSH release activity. Although studies with naloxone (another opioid antagonist) have shown reduced food intake in rats, studies with naltrexone have been disappointing, as it led to minimal or no weight loss as a monotherapy (145).

The idea of associating an opioid receptor antagonist to block the autoinhibitory feedback in POMC neurons of the ARC emerged as a good strategy to enhance the anorectic effect of bupropion. This led to the development of the fixed-dose combination of naltrexone and bupropion. The fixed-dose combination of naltrexone 8 mg and bupropion 90 mg (Contrave^®^) has a synergistic effect (145).

### 5.2 Dosage/usage instructions

The dosage of the N/B combination should be titrated weekly. The starting dose is one tablet in the morning for 7 days, followed by a progressive increase to one tablet every 12 hours in the second week, two tablets in the morning and one tablet at night in the third week, and two tablets every 12 hours from the fourth week onward. Tablets should not be broken, chewed, or crushed, and total daily doses exceeding 32 mg/360 mg per day are not recommended. The tablet can be administered with meals but should not be taken with high-fat meals due to a significant increase in systemic exposure to bupropion and naltrexone (146).

### 5.3 Tolerability and side effects

The most common adverse events, affecting over 4% of the individuals who used this medication, were nausea (32.5%), constipation (19.2%), vomiting (17.6%), and headache (10.7%), as well as dizziness, insomnia, xerostomia, diarrhea, anxiety, hot flushes, fatigue, and tremor (146).

A meta-analysis evaluating study discontinuation due to adverse effects of antiobesity agents included four studies assessing the N/B combination. Out of 2,044 participants in the N/B group, 501 had adverse events compared with 175 of 1,319 in the placebo group (OR = 2.6) (147). Table 2 describes the SAEs and discontinuation rates observed in the phase 3 studies. The most frequent adverse reactions leading to discontinuation were nausea (6.3%), headache (1.7%), and vomiting (1.1%).

**Table 2:** Description of severe adverse events and discontinuation rates in phase 3 studies of the naltrexone/bupropion combination

Study	Incidence of SAE		Discontinuation rates	
	Intervention	Control	Intervention n/N	Control n/N
COR-I (148)	SAE 1.6%: 1 fatal myocardial infarction and 1 heart failure (both considered not DR)	1 pericardial effusion	287/583	291/581
COR-II (149)	SAE 2%: 1 myocardial infarction, 1 seizure	SAE 1.4%	461/1,001 241 AE	226/495 68 AE
COR-BMOD (150)	Two cholecystitis (possibly DR), 0 suicidal ideations	2 suicidal ideations, 0 cholecystitis	249/591	84/202
COR-DM (151)	NS 3.9%	NS 4.7%	160/335	70/170

AE: adverse event; SAE: severe adverse event; n/N: number of discontinuations/total number of study participants; NS: not specified; DR: drug-related.

### 5.4 Absolute contraindications

The combination of N/B is contraindicated in the following clinical conditions: uncontrolled hypertension, epilepsy or history of seizures, severe hepatic impairment, grade 5 CKD, presence of CNS tumor, history of bipolar disorder, bulimia or anorexia nervosa (increased risk of seizures), chronic use of opioid or opiate agonists or partial agonists or acute withdrawal of opiates, abrupt discontinuation of alcohol, benzodiazepines, barbiturates and antiepileptic drugs, concomitant administration of monoamine oxidase inhibitors (MAOIs; a gap of at least 14 days must be given between discontinuation of MAOI and treatment initiation), and known allergy to bupropion or naltrexone (146).

The N/B combination should be suspended 24-72 hours before small- and medium-size surgeries and 72 hours before major surgeries or procedures requiring intensive pain management with opioids to eliminate the antagonistic effect of the medication on opioid analgesia, while bupropion should be continued. It is recommended to reintroduce N/B 7 days after cessation of opioids in the postoperative period.

### 5.5 Efficacy

#### 5.5.1 Efficacy of bupropion/naltrexone on body weight

The weight loss and categorical weight loss percentages of 5% and 10% found in the main studies are summarized in Table 3. The clinical development program for the N/B combination was named Contrave Obesity Research (COR) and involved two phase 2 studies and four phase 3 studies: COR-I (148), COR-II (149), COR-BMOD (Behavior Modification) (150), and COR-Diabetes (151).

**Table 3:** Main studies evaluating the naltrexone/bupropion combination in obesity treatment

Publication	Participant characteristics and publication	Weight loss subtracted from placebo	Weight loss ≥ 5%	Weight loss ≥ 10%	Weight loss ≥ 15%
Greenway and cols. (N = 238) (152)	BMI 30-40 kg/m^2^, 16 weeks	-3%	-	-	-
Greenway and cols. (N = 419) (153)	BMI 30-40 kg/m^2^, 24 weeks (N/B 32/400 mg)	-4.65%	-	-	-
COR-I (N = 1,742) (148)	BMI 30-40 kg/m^2^ without comorbidities or BMI 27-29.9 kg/m^2^ with dyslipidemia or hypertension, 56 weeks	-4.8%	62%	34%	17%
COR-BMOD (N = 793) (150)	BMI 30-40 kg/m^2^ without comorbidities or BMI 27-29.9 kg/m^2^ with dyslipidemia or hypertension, 56 weeks	-4.2%	80.4%	55.2%	39.5%
COR-II (N = 1,496) (149)	BMI 30-40 kg/m^2^ without comorbidities or BMI 27-29.9 kg/m^2^ with dyslipidemia or hypertension, 28 weeks	-4.6%	64.9%	39.4%	18.9%
COR-DM (N = 505) (151)	BMI 27-40 kg/m^2^ with T2DM, 56 weeks	-2.8%	53.1%	26.3%	NA

* All studies used naltrexone/bupropion (N/B) 32/360 mg unless otherwise noted.

BMOD: Behavior Modification; COR: Contrave Obesity Research; BMI: body mass index; NA: not available in the publication.

#### 5.5.2 Effects on body composition

In a 24-week phase 2 study comparing placebo, naltrexone monotherapy, bupropion monotherapy, and one of three N/B dose combinations for efficacy and safety, a subgroup underwent body composition analysis using DXA and computed tomography. Eighty participants completed this subgroup analysis. The N/B combination resulted in weight loss and greater reduction in body fat (-14.0 ± 1.3%) than placebo (-4.0 ± 2.0%), naltrexone monotherapy (-3.2 ± 2.5%), and bupropion monotherapy (-4.1 ± 2.9%; all p < 0.01). The reduction in visceral adipose tissue mass was also greater with N/B (-15.0 ± 1.8%) than with placebo (-4.6 ± 2.7%), naltrexone monotherapy (-0.1 ± 3.5%), and bupropion monotherapy (-2.3 ± 4.2%; all p < 0.01). The reductions in body fat and visceral adipose tissue mass with N/B were proportional to weight loss, and weight loss with N/B was not associated with a greater relative reduction in lean mass than placebo or monotherapies (154).

#### 5.5.3 Effects on glycemic control in patients with type 2 diabetes mellitus

The COR-Diabetes study evaluated patients with T2DM who did not achieve the glycemic goal of HbA1c level < 7% with oral antidiabetic agents or with diet and exercise alone. In the entire population of these four studies, 24% of participants had hypertension and 54% had dyslipidemia at baseline (151). Table 4 presents the main results of the COR-Diabetes study.

**Table 4:** Variation in glycemic parameters in patients with type 2 diabetes mellitus treated with the naltrexone/bupropion combination in the COR-Diabetes study

	N/B combination (n = 265)		Placebo (n = 159)		N/B minus placebo (mean)
	Baseline	Change from baseline (mean)	Baseline	Change from baseline (mean)	
HbA1c (%)	8.0	-0.6	8.0	-0.1	-0.5*
Fasting blood glucose (mg/dL)	160.0	-11.9	163.9	-4.0	-7.9

Based on the last observation carried forward (LOCF) during the COR-DM study.

* Statistically significant *versus* placebo (p < 0.001).

#### 5.5.4 Effects of bupropion/naltrexone in patients with prediabetes/glucose intolerance

The effects of the N/B combination in patients with prediabetes/glucose intolerance have not been evaluated.

#### 5.5.5 Effects of bupropion/naltrexone on lipid metabolism

In the COR-Diabetes (151) study, which evaluated patients with T2DM outside the HbA1c target, 54% had dyslipidemia at baseline. Compared with placebo, participants treated with N/B had a mean reduction of 11.2% in triglycerides (*versus a* reduction of 0.8% in the placebo group) and an increase of 3.0 ± 0.5 mg/dL in HDL-c (*versus a* reduction of 0.3 ± 0.6 mg/dL in the placebo group), with no significant effect on LDL-c. The magnitude of these variations in the COR-I (148) and COR-BMOD (150) studies was similar.

#### 5.5.6 Effects of bupropion/naltrexone on blood pressure and heart rate

The N/B combination may elevate SBP and/or DBP values and increase resting heart rate. Both BP and pulse should be measured prior to therapy initiation with the N/B combination and monitored at regular intervals consistent with usual clinical practice, particularly in patients with controlled hypertension prior to treatment. The N/B combination should not be administered to patients with uncontrolled hypertension. Among patients treated with the N/B combination in placebo-controlled clinical studies, mean SBP and DBP values were approximately 1 mmHg above those at baseline at weeks 4 and 8, similar to those at baseline at week 12, and approximately 1 mmHg below those at baseline between weeks 24 and 56. In contrast, among patients treated with placebo, the mean BP value was approximately 2-3 mmHg below the baseline value across the same time points, yielding statistically significant differences between groups at all assessments during this period. The largest mean differences between the groups were observed in the first 12 weeks (treatment difference +1.8 to +2.4 mmHg for SBP; +1.7 to +2.1 mmHg for DBP) (148-153).

#### 5.5.7 Effects on obstructive sleep apnea syndrome

The effects of the N/B combination in patients with OSAS have not been evaluated.

#### 5.5.8 Effects of bupropion/naltrexone in patients with polycystic ovary syndrome

The effects of the N/B combination in women with PCOS have not been evaluated.

#### 5.5.9 Effects of bupropion/naltrexone in patients with male hypogonadism

The effects of the N/B combination in men with hypogonadism have not been evaluated.

#### 5.5.10 Effects of bupropion/naltrexone on metabolic dysfunction-associated steatotic liver disease

There are limited data on the effects of the N/B combination in patients with MASLD. In a *post hoc* analysis of four RCTs, the N/B combination for 1 year resulted in an improvement in fibrosis-4 index (FIB-4; a noninvasive index of liver fibrosis) independent of potential confounders, including weight change. The effect of N/B intervention was independently associated with a decrease in ALT (155).

#### 5.5.11 Effects of bupropion/naltrexone on quality of life

The N/B combination was evaluated in a multicenter, randomized, controlled, open-label study examining weight-related quality of life, control over eating behavior, and sexual function after 26 weeks of treatment plus a comprehensive LSC program (N/B + LSC, n = 153) or usual care (UC, n = 89), which included minimal lifestyle intervention.

Participants in the N/B + LSC group and UC group lost, respectively, 9.46% and 0.94% of their initial body weight at week 26 (p < 0.0001). The participants in the N/B + LSC group had greater improvement in the total IWQOL score compared with those in the UC group (p < 0.0001). Among participants with moderate/severe scores on the binge eating scale, 91% of N/B + LSC participants *versus* 18% of UC participants experienced improvement. In participants with sexual dysfunction defined by the Arizona Sexual Experiences Scale, 58% of N/B + LSC participants and 19% of UC participants no longer met the criteria for dysfunction at week 26 (156).

#### 5.5.12 Effects of bupropion/naltrexone on osteoarticular diseases

The effects of the N/B combination in patients with osteoarthritis or other osteoarticular diseases have not been evaluated.

#### 5.5.13 Effects of bupropion/naltrexone in patients with renal disease

The effects of the N/B combination in patients with renal disease have not been evaluated.

#### 5.5.14 Effects of bupropion/naltrexone on cardiovascular diseases

The LIGHT study was designed to determine the cardiovascular safety of N/B compared with placebo in patients with overweight or obesity. The trial enrolled 8,910 patients with overweight or obesity who had increased cardiovascular risk, but after public disclosure by the sponsor of confidential interim data during the trial, the study’s academic leadership recommended termination of the trial, which was agreed by the sponsor. Male participants were older than 45 years and female participants were older than 50 years, and the mean age was 61.0 ± 7.3 years. For the 25% interim analysis, cardiovascular outcomes occurred in 59 patients treated with placebo (1.3%) and in 35 patients treated with N/B (0.8%; HR = 0.59). After 50% of planned events, cardiovascular outcomes occurred in 102 patients (2.3%) in the placebo group and in 90 patients (2.0%) in the N/B group (157).

## 6. TIRZEPATIDE

At the time this document was prepared, tirzepatide was only approved in Brazil for the treatment of patients with T2DM (September 2023). However, it has already been approved in Europe (April 2024) and in the United States (November 2023) for the treatment of obesity. The application for approval has already been submitted to Anvisa, and the authors of the present document believe that approval in Brazil should be obtained in the near future. Until approval is granted, the use of tirzepatide for the treatment of obesity in Brazil will be considered off-label.

### 6.1 Mechanism of action

Tirzepatide, the first medication in the incretin class with a dual mechanism of action, is a synthetic peptide with dual agonism action on GLP-1 and glucose-dependent insulinotropic polypeptide (GIP) receptors. Notably, GIP is a peptide secreted by K cells in the duodenum and jejunum in response to nutrient intake. It regulates energy balance through cell surface receptor signaling in CNS cells and adipose tissue (158).

Engineered from the native GIP sequence, preclinical data show a proportionally higher affinity of tirzepatide for GIP receptors compared with GLP-1 receptors (1:5). The GIP receptor activation appears to act synergistically with GLP-1 receptor activation to yield a greater weight reduction in mice than that achieved with GLP-1 receptor monoagonism (158).

The exact molecular mechanisms involved in the therapeutic effects of tirzepatide on glycemic control and body weight are not yet fully understood. One hypothesis is that GLP-1 activity reduces glucose levels, facilitating the effects of GIP on resensitized beta cells. Tirzepatide also appears to act as a more potent coagonist compared with GLP-1, with little β-arrestin recruitment and receptor internalization, which could explain its superior activity in target cells (159).

### 6.2 Dosage/usage instructions

The initial dose of tirzepatide to begin titration is 2.5 mg applied subcutaneously once weekly. After 4 weeks, the dose should be increased to 5 mg. Increases of 2.5 mg can be made every minimum period of 4 weeks, reaching a maximum once-weekly dose of 15 mg. Based on the pharmacokinetics of tirzepatide, no dose adjustment is recommended based on age, gender, or body weight or in patients with hepatic or renal impairment (including those with ESRD) (160).

### 6.3 Tolerability/side effects

In the SURMOUNT-1 study, the most common adverse events were gastrointestinal in nature. Nausea was the most frequent side effect, observed in 24.6%-31% of patients, mainly during the dose titration period. Other reported effects were diarrhea and constipation (23% and 11.7%, respectively), all with mild-to-moderate severity, causing treatment discontinuation in a maximum of 7.1% of patients (161).

Tirzepatide, at doses of 5 to 15 mg, was well tolerated during the SURPASS program: SAEs were reported in 1%-8% of participants with diabetes (SURPASS 1-3) (162-164) and in 6%-17% of participants with more advanced diabetes (SURPASS 4-5) (165,166) – these SAE rates are similar to those reported in placebo and active comparator groups.

The incidence of gastrointestinal adverse events was similar between tirzepatide, semaglutide, and dulaglutide. Most adverse events were mild to moderate, dose-dependent, and occurred during dose escalation and subsequent reduction.

### 6.4 Absolute contraindications

The use of tirzepatide is contraindicated during pregnancy and in patients with a personal history of chronic pancreatitis or a personal or family history of medullary thyroid cancer or multiple endocrine neoplasia 2A or 2B.

### 6.5 Efficacy

#### 6.5.1 Efficacy of tirzepatide on body weight

The phase 3 SURMOUNT-1 RCT compared the response to weekly tirzepatide at doses of 5 mg, 10 mg, or 15 mg *versus* placebo in 2,539 adults with obesity or BMI > 27 kg/m^2^associated with at least one weight-related complication, excluding diabetes. The follow-up duration was 72 weeks, including the 20-week dose-escalation period (161). In this study, the average initial weight was 104.8 kg, and BMI was 38 kg/m^2^. The mean reduction in body weight observed at week 72 with tirzepatide was 16.0% (16.8%-15.2%) with the 5 mg dose, 21.4% (22.2%-20.6%; which was equivalent to 22.2 kg body weight reduction) with the 10 mg dose, and 20.9% (21.9%-19.9% or 23.6 kg) with the 15 mg dose.

The SURMOUNT-2 RCT evaluated treatment with subcutaneous tirzepatide (10 mg or 15 mg) once weekly or placebo for 72 weeks in 1,514 adults with obesity and T2DM. The primary outcomes were the percent change in body weight from baseline and body weight reduction of 5% or more. At baseline, the mean body weight was 100.7 kg (standard deviation ± 21.1 kg), BMI was 36.1 kg/m^2^ (±6.6 kg/m^2^), and HbA1c level was 8.02% (±0.89%). The mean changes in body weight at week 72 with tirzepatide 10 mg and 15 mg were -12.8% (±0.6%) and -14.7% (±0.5%), respectively, and -3.2% (±0.5%) with placebo, resulting in estimated treatment differences *versus* placebo of -9.6% (95% CI = -11.1 to -8.1%) with tirzepatide 10 mg and -11.6% (95% CI = -13.0 to -10.1%) with tirzepatide 15 mg (all p < 0.0001) (167).

The SURMOUNT-3 RCT evaluated the impact of tirzepatide in individuals with obesity who had an adequate response to treatment with intensive LSCs. It included 579 individuals with BMI > 30 kg/m^2^ or 27 kg/m^2^ (with at least one comorbidity associated with obesity) who achieved a minimum weight loss of 5% after 12 weeks of intensive LSCs. After randomization, patients receiving tirzepatide for 72 weeks had a mean weight change of -18.5% compared with -2.5% in the placebo group (168).

Table 5 highlights the categorical weight loss observed in the SURMOUNT series studies.

**Table 5:** Categorical weight loss with weekly tirzepatide 10 mg or 15 mg in the SURMOUNT series of studies, according to the percentage of weight lost

Study/duration	Population	Loss > 5%	Loss > 10%	Loss > 15%	Loss > 20%
SURMOUNT-1 (72 weeks) (161)	Individuals with overweight or obesity and without T2DM	90.9%*	83.5%*	70.6%*	56.7%*
SURMOUNT-2 (72 weeks) (167)	Individuals with T2DM, with BMI > 27 kg/m^2^ and HbA1c between 7.0% and 10%	79/83%**	61/65%**	40/48%**	22/31%**
SURMOUNT-3 (72 weeks) (168)	Same as SURMOUNT-1; after 12 weeks, patients who achieved > 5% weight reduction with intensive lifestyle change were randomized to placebo or tirzepatide	87.5%	76.7%	65.4%	44.7%
SURMOUNT-4 (36 weeks open-label + 52 weeks) (169)	Same as SURMOUNT-1; after 36 weeks, patients on tirzepatide 10 mg or 15 mg were randomized to placebo or tirzepatide	97.3%	92.1%	84.1%	69.5%

* Tirzepatide 15 mg/week.

** Tirzepatide 10 mg/15 mg.

#### 6.5.2 Effects of tirzepatide on weight maintenance

The effects of tirzepatide on weight maintenance were evaluated in the SURMOUNT-4 RCT. This study enrolled 783 participants in an initial 36-week open-label period who received tirzepatide 10 mg or 15 mg. At week 36, a total of 670 participants were randomized to continue treatment with tirzepatide (n = 335) or switch to placebo (n = 335) for an additional 52 weeks. In the initial 36-week period, participants (mean initial weight 107.3 kg) lost an average of 20.9% of their body weight. From weeks 36 to 88, participants who remained on tirzepatide had an average additional weight loss of 5.5%, while the group randomized to placebo gained an average of 14.0%. In conclusion, withdrawal of tirzepatide led to a substantial regain of lost weight, while the continuation of the medication not only maintained the weight lost but also led to an additional weight loss (169).

#### 6.5.3 Effects of tirzepatide on body composition

In the SURMOUNT-1 study, a subgroup of 160 participants underwent body composition analysis using DXA. The results showed greater fat mass reduction in the tirzepatide group compared with the placebo group (33.9% *versus* 8.2%, respectively, difference -25.7%). Similarly, the ratio between total fat mass and lean mass reduced more in the tirzepatide group (from 0.93 to 0.70) than in the placebo group (from 0.95 to 0.88), from baseline to week 72 (161).

A plethysmography analysis was conducted to compare body composition changes in 45 individuals with T2DM treated with tirzepatide 15 mg/week, 44 treated with semaglutide 1 mg/week, and 28 treated with placebo. At week 28, the tirzepatide-treated group experienced greater fat mass reduction than the placebo group (9.6 kg [12.4 to 6.9 kg]; p < 0.001) and semaglutide group (3.8 kg; p < 0.002). Similarly, the reduction in FFM was greater in the tirzepatide group compared with the placebo group (1.5 kg; p < 0.001) and semaglutide group (0.8 kg; p < 0.018) (170).

#### 6.5.4 Effects of tirzepatide in patients with prediabetes/glucose intolerance

In the SURMOUNT-1 study, 95.3% of individuals with prediabetes at baseline reverted to normoglycemia with tirzepatide, compared with 61.9% in the placebo group (161). Treatment with tirzepatide significantly reduced the 10-year predicted risk of T2DM development compared with placebo in participants with obesity or overweight, independent from baseline glycemic status. This was the finding of a *post hoc* analysis of the SURMOUNT-1 study, which used a cardiometabolic disease staging risk score to calculate the predicted 10-year risk of T2DM at baseline and at study weeks 24 and 72. At week 72, the mean absolute predicted risk score reductions for T2DM were significantly greater in the tirzepatide groups (5 mg, 12.4%; 10 mg, 14.4%; 15 mg, 14.7%) compared with the placebo group (0.7%). Participants with prediabetes had greater mean reductions in risk score from baseline (16.0%-20.3%) compared with those without prediabetes (10.1%-11.3%) (171).

#### 6.5.5 Effects of tirzepatide in patients with type 2 diabetes mellitus

A recent meta-analysis evaluated 6,609 individuals with T2DM included in seven RCTs lasting at least 12 weeks to analyze the efficacy of different tirzepatide doses (5 mg, 10 mg, and 15 mg) in reducing HbA1c levels compared with other antidiabetic agents or placebo. Tirzepatide was superior in reducing HbA1c levels in a dose-dependent manner, with mean differences ranging from -1.62% to -2.06% *versus* placebo, -0.29% to -0.92% *versus* GLP-1as, and -0.70% to -1.09% *versus* basal insulin regimens (172).

The SURPASS-2 study included 1,876 patients with T2DM and compared tirzepatide 5 mg, 10 mg, and 15 mg *versus* semaglutide 1 mg in a 1:1:1:1 design for 40 weeks, with the primary outcome of reduction in HbA1c level. The mean reductions in HbA1c levels were 2.01%, 2.24%, and 2.30% with tirzepatide 5 mg, 10 mg, and 15 mg, respectively, and 1.86% with semaglutide 1.0 mg. The baseline mean HbA1c level was 8.28%. After 40 weeks, almost half of the patients who received tirzepatide 10 mg and 15 mg (40% and 46%, respectively) had HbA1c levels ≤ 5.7%. This was observed in 27% of the patients who received tirzepatide 5 mg and in 19% of those who received semaglutide 1 mg (163).

#### 6.5.6 Effects of tirzepatide on lipid metabolism

In the SURPASS 1 to 5 study programs, treatment with tirzepatide at doses of 5 mg, 10 mg, and 15 mg resulted in reductions in serum triglyceride and LDL-c levels (162-166,173).

#### 6.5.7 Effects of tirzepatide on blood pressure and heart rate

In the SURPASS 1 to 5 program studies, tirzepatide treatment of patients with T2DM resulted in mean reductions in SBP and DBP values of 6-9 mmHg and 3-4 mmHg, respectively. There was a mean reduction in SBP and DBP of 2 mmHg each in patients treated with placebo. In placebo-controlled phase 3 studies, treatment with tirzepatide resulted in a mean heart rate increase of 2-4 bpm compared with a mean heart rate increase of 1 bpm with placebo (162-166). In the SURMOUNT-1 study, individuals with obesity/overweight without diabetes had mean reductions of 7.2 mmHg in SBP and 4.8 mmHg in DBP with tirzepatide compared with mean reductions of 1 mmHg and 0.8 mmHg, respectively, with placebo (161).

#### 6.5.8 Effects of tirzepatide on obstructive sleep apnea syndrome

A 52-week RCT (SURMOUNT-OSA) was conducted to evaluate the efficacy and safety of tirzepatide at the maximum tolerated dose (10 mg or 15 mg) *versus* placebo as an adjunct to diet and exercise in participants with moderate-to-severe OSAS (AHI ≥ 15). Patients treated with tirzepatide (10 mg or 15 mg weekly) experienced an AHI reduction of 27.4 events/hour compared with 4.8 events/hour in those treated with placebo. As a secondary outcome, tirzepatide led to a mean AHI reduction of 55% compared with 5.0% with placebo. Finally, the mean weight loss was 18.1% in the tirzepatide group compared with 1.3% in the placebo group (174).

#### 6.5.9 Effects of tirzepatide in patients with polycystic ovary syndrome

Tirzepatide has not been evaluated for effects in women with PCOS.

#### 6.5.10 Effects of tirzepatide in patients with male hypogonadism

Tirzepatide has not been evaluated for effects in patients with male hypogonadism.

#### 6.5.11 Effects of tirzepatide on nonalcoholic fatty liver disease

A study used magnetic resonance imaging to evaluate the liver fat content, volume of visceral adipose tissue, and abdominal subcutaneous adipose tissue in 296 individuals with T2DM treated with tirzepatide or insulin degludec participating in the SURPASS-3 study. At week 52, the participants using tirzepatide (pooled tirzepatide 10 mg and 15 mg groups) experienced significantly greater mean reductions in liver fat content compared with those using insulin degludec (-8.1% *versus* -3.4%), respectively, from a baseline liver fat content of 15.7% and 16.6%, respectively (175).

At 52 weeks, participants treated with tirzepatide 5 mg, 10 mg, and 15 mg had significantly greater reductions in volume of visceral adipose tissue (-1.10 L, -1.53 L, and -1.65 L, respectively) and abdominal subcutaneous adipose tissue (-1.40 L, -2.25 L, and -2.05 L, respectively) compared with their respective baseline values of 6.6 L and 10.4 L. These reductions contrasted with the increases observed in the insulin degludec-treated group (0.38 L and 0.63 L) (175). Overall, 67%-81% of tirzepatide-treated participants achieved at least a 30% reduction in liver fat content.

Another *post hoc* analysis evaluated the effects of tirzepatide on MASLD and fibrosis biomarkers in patients with T2DM compared with dulaglutide and placebo for 26 weeks and showed that the higher dose of tirzepatide significantly decreased MASLD-related biomarkers and increased adiponectin in these patients (176).

A phase 2 RCT was conducted to evaluate the effects of tirzepatide treatment in individuals with biopsy-confirmed MASH and stage F2 or F3 fibrosis. The patients were randomized to placebo or tirzepatide 5 mg, 10 mg, or 15 mg (n = 190) and treated for 52 weeks, when the biopsy was then repeated. The percentage of patients who achieved the MASH improvement endpoint without fibrosis progression was 10% in the placebo group, 44% in the tirzepatide 5 mg group, 56% in the tirzepatide 10 mg group, and 62% in the tirzepatide 15 mg group. The percentage of patients who had improvement in at least one fibrosis stage (without worsening of MASH) was 30% in the placebo group, 55% in the tirzepatide 5 mg group, 51% in the tirzepatide 10 mg group, and 61% in the tirzepatide 15 mg group (177).

#### 6.5.12 Effects of tirzepatide on quality of life

An exploratory analysis of the phase 3 SURPASS J-mono study assessed treatment satisfaction using the Japanese translation of the Diabetes Treatment Satisfaction Questionnaire (DTSQs) and the DTSQc version. After 52 weeks of treatment, there was a trend toward greater satisfaction among patients who received any dose of tirzepatide compared with those who received dulaglutide. The overall mean DTSQc scores at week 52 were significantly higher with tirzepatide 5 mg, 10 mg, and 15 mg *versus* dulaglutide 0.75 mg (11.5, 12.1, and 12.3, respectively, *versus* 8.9; p < 0.001). *Post hoc* subgroup analyses demonstrated greater treatment satisfaction with tirzepatide compared with dulaglutide in the subgroup with ages below 65 years (p < 0.001) and baseline BMI ≥ 25 kg/m^2^ (p < 0.01), along with similar treatment satisfaction across treatment arms in the subgroup with ages 65 years or above and with BMI < 25 kg/m^2^ (178).

#### 6.5.13 Effects of tirzepatide on osteoarticular diseases

Tirzepatide has not been evaluated for effects in osteoarticular diseases.

#### 6.5.14 Effects of tirzepatide in patients with chronic kidney disease

An exploratory *post hoc* analysis of SURPASS-4 showed that tirzepatide reduced the decline in eGFR and decreased the urine albumin-to-creatinine ratio (UACR) compared with insulin glargine in individuals with T2DM and high cardiovascular risk. At baseline, participants had a mean eGFR of 81 mL/min/1.73 m^2^ and median UACR of 15 mg/g (17% of participants had eGFR < 60 mL/min/1.73 m^2^, 28% had microalbuminuria, and 8% had macroalbuminuria). The mean rate of eGFR decline was -1.4 mL/min/1.73 m^2^ per year for the combined tirzepatide treatment groups *versus* -3.6 mL/min/1.73 m^2^ per year in the insulin group. The UACR increased from baseline with insulin glargine (36.9%) but not with tirzepatide (-6.8%), with a between-group frequency difference of -31.9%. Participants receiving tirzepatide had fewer occurrences of the composite renal outcome (time to first occurrence of eGFR decline of at least 40% from baseline, ESRD, death due to renal failure, or new-onset macroalbuminuria) compared with those receiving insulin glargine (HR = 0.58; 95% CI = 0.43-0.8). These findings were primarily driven by a reduced number of individuals developing new-onset macroalbuminuria (179).

#### 6.5.15 Effects of tirzepatide on cardiovascular diseases

A meta-analysis of cardiovascular outcomes included seven RCTs with at least 26 weeks of follow-up comparing the time to occurrence to the first prespecified major adverse cardiac event (MACE; including cardiovascular death, AMI, stroke, and hospitalization for unstable angina) between participants using combined doses of tirzepatide (n = 4,887) and controls (n = 2,328). One-third of the participants had established CVD. In all, 142 participants experienced at least one MACE event after treatment for just over 1 year. The HRs comparing tirzepatide *versus* control were 0.80 (95% CI = 0.57-1.11) for MACE-4 (*i.e.*, the four major adverse cardiac events considered in the trial), 0.90 (95% CI = 0.50-1.61) for cardiovascular death, and 0.80 (95% CI = 0.51-1.25) for all-cause death (180). These results suggest that tirzepatide does not increase cardiovascular risk. However, the exact impact of tirzepatide on cardiovascular outcomes in individuals with T2DM and established CVD will be addressed in the SURPASS-CVOT trial, an ongoing study evaluating the noninferiority and superiority of tirzepatide *versus* dulaglutide 1.5 mg for cardiovascular safety in individuals with T2DM and atherosclerosis confirmed by prior CVD (ClinicalTrials.gov Identifier: NCT04255433).

Table 6 presents the effects of the different medications approved for treating obesity in Brazil after an average treatment period of 1 year. Differences in methodology and statistical analysis among the studies hinder a direct comparison between the medications.

**Table 6:** Efficacy of 1-year treatment of individuals with obesity using medications approved for treating obesity in Brazil

			Percentage achieving weight loss goals in 1 year with medication					
Medication	Starting weight (kg)	1 year*	>5%		>10%	>15%	>20%	>25%
Sibutramine 10 mg (181)	-	-4.4 kg (SIB)/-1.6 kg (PLB)/NA	39		7	NA	NA	NA
Sibutramine 15 mg (181)	-	-6.4 kg (SIB)/-1.6 kg (PLB)/NA	57		34	NA	NA	NA
Orlistat 120 mg (42)	99.1 (61.0-148.6)	-3.9 kg/NA%	68.5	29.5 (loss between 10.1 and 20.0%)			9.3	NA
Liraglutide 3.0 mg (52)	106.2 ± 21.2	-5.6 kg/-5.4%	63.2		33.1	14.4	NA	NA
Semaglutide 2.4 mg (119)	105.4 ± 22.1	-12.7 kg/-12.4%	86.4		69.1	50.5	32	NA
*Tirzepatide 10 mg (161)	105.6 ± 22.92	NA kg/-16.4%	88.9		78.1	66.6	50.1	32.3
*Tirzepatide 15 mg (161)	105.6 ± 22.92	NA kg/-17.8%	90.9		83.5	70.6	56.7	36.2
Naltrexone/bupropion (N/B) 32/360 mg (148)	99.7 ± 15.9	-8.1% (N/B)/-1.8% (PLB)** -8.0 kg (N/B)/-1.9 kg (PLB)***	62		34	17	NA	NA

Note: data are presented as mean ± standard deviation, except for orlistat, for which they are presented as median (minimum – maximum).

NA: information not available in the original publication; SIB: sibutramine; PLB: placebo.

* Difference in weight loss in the treatment group *versus* the placebo group. ** Completers. *** Difference in weight loss in the treatment group *versus* the placebo group not available in the article.

In conclusion, historically, pharmacological treatments for obesity have been underutilized, with very few drug options available for a long time. Fortunately, this landscape is changing rapidly. In recent years, several new drugs with varying mechanisms of action, efficacy, and safety profiles (see Table 7 for summary) have emerged in Brazil. This document aims to provide a comprehensive literature review of the available pharmacological options with out establishing a definitive guideline, which is expected to be published in the near future. The goal is to familiarize healthcare providers with these options, whether they prescribe them as medical doctors or simply receive patients in use (who could need guidance) or refer them to treatment. We hope this document can serve as an useful guide and also a tool to reduce stigma surrounding obesity pharmacology.

**Table 7:** Most common and specific side effects of the anti obesity pharmacologic agents

	More than 10% of patients	Specific side effects that deserve attention
Sibutramine 10-15 mg	Constipation, xerostomia, insomnia	Tachycardia/increased heart rate, increased blood pressure, headache, anxiety
Orlistat 120 mg 3 x/day	Diarrhea/steatorrhea/urgency, flatulence, upper respiratory tract infections/flu, headache, hypoglycemia	Hypersensitivity reactions, long-term deficiency of fat-soluble vitamins
Liraglutide 3.0 mg/day	Nausea and vomiting, diarrhea, constipation	Injection site reactions, increased heart rate, insomnia, cholelithiasis, asthenia and fatigue, hypoglycemia
Semaglutide 2.4 mg/week	Nausea and vomiting, diarrhea, constipation, abdominal pain, headache, fatigue	Injection site reactions, increased heart rate, cholelithiasis, hypoglycemia
*Tirzepatide 10 and 15 mg/week	Hypoglycemia (when used with sulfonylureas or insulin), nausea, diarrhea	Hypersensitivity reactions, increased heart rate, injection site reactions
Naltrexone/Bupropion 360/32 mg/day	Nausea, constipation, headache, vomiting	Suicidal thoughts or actions, seizures, risk of opioid overdose, sudden opioid withdrawal, severe allergic reactions, increased blood pressure or heart rate, hepatitis, manic episodes, narrow-angle glaucoma, hypoglycemia (when used with sulfonylureas or insulin), serotonin syndrome

*As of July 2024, tirzepatide was not yet approved to treat obesity in Brazil. Up to this date, the medication has been approved ONLY to treat type 2 diabetes mellitus.




