# Supplementary material for: Pharmacologic Treatment of Obesity in adults and its impact on comorbidities: 2024 Update and Position Statement of Specialists from the Brazilian Association for the Study of Obesity and Metabolic Syndrome (Abeso) and the Brazilian Society of Endocrinology and Metabolism (SBEM)
Source: Arch Endocrinol Metab. 2024 Nov 25;68:e240422. doi: 10.20945/2359-4292-2024-0422 (PMC11634287; doi:10.20945/2359-4292-2024-0422)
Supplement: Supplementary file 1 [file 2359-4292-aem-68-e240422-suppl01.pdf]

Chart 1 displays a color map summarizing the main effects of medications approved for treating obesity. It is important to note that the color map represents the authors' opinions in this document regarding the available evidence and does not adhere to any specific degree of recommendation or level of evidence.

Chart 1. Summary of the main effects of medications approved for the treatment of obesity in Brazil

	Sibutramine 10-15 mg/day	Orlistat 120 mg 3 x/day	Liraglutide 3.0 mg/day	Semaglutide 2.4 mg/wk	**Tirzepatide 10 and 15 mg/ week	Naltrexone/ bupropion 32/360 mg/day
**Prediabetes**	Blue	Dark green	Dark green	Dark green	Dark green	Blue
**T2DM (glycemic control)**	Dark green	Dark green	Dark green	Dark green	Dark green	Dark green
**Lipid profile**	Dark green	Dark green	Dark green	Dark green	Dark green	Dark green
**BP**	Yellow	Dark green	Dark green	Dark green	Dark green	Yellow
**OSAS**	Light green	Light green	Dark green	Blue	Dark green	Blue
**PCOS**	Light green	Dark green	Light green	Light green	Blue	Blue
**Male hypogonadism**	Light green	Blue	Light green	Blue	Blue	Blue
**MASLD**	Light green	Light green	Dark green	Dark green	Dark green	Light green
**Osteoarticular diseases**	Blue	Dark green	Blue	Dark green	Blue	Blue
**CKD**	Blue	Blue	Light green	Dark green	Light green	Blue
**CVD***	Red	Blue	Blue	Dark green	RCT in progress	RCT in progress
**HF**	Red	Blue	Blue	Dark green	Blue	Blue
Quality of life	Dark green	Dark green	Dark green	Dark green	Dark green	Dark green

Dark green: well-designed clinical trials or high-quality systematic reviews/meta-analyses of clinical trials.
Light green: clinical trials with few participants, observational design, or low statistical power.
Yellow: clinical trials with adverse events that deserve attention/caution.
Red: clinical trials with evidence of risk for patients at high cardiovascular risk.
Blue: no studies available on the topic or studies with neutral results (no benefits demonstrated).
T2DM: type 2 diabetes mellitus; BP: blood pressure; OSAS: obstructive sleep apnea syndrome; PCOS: polycystic ovary syndrome; MASLD: metabolic dysfunction-associated steatotic liver disease; CKD: chronic kidney disease; CVD: cardiovascular disease; RCT: randomized clinical trial; HF: heart failure.
* For cardiovascular diseases, only randomized clinical trials designed specifically to assess safety and/or superiority in cardiovascular outcomes were considered.
Prepared according to the authors' opinions.
**As of July 2024, tirzepatide was not yet approved for the treatment of obesity in Brazil. Up to this date, the medication was ONLY approved to treat type 2 diabetes mellitus.

Copyright© AE&M all rights reserved.
